# Generating Ultra‐Fast Protein *trans*‐Splicing of a Cysteine‐Less and Semisynthetic Split Intein for Chemical Protein Labeling

**DOI:** 10.1002/cbic.202500969

**Published:** 2026-03-24

**Authors:** Christoph Humberg, Tobias M. E. Terhorst, Tim Pasch, Henning D. Mootz

**Affiliations:** ^1^ Department of Chemistry and Pharmacy Institute of Biochemistry University of Münster Münster Germany

**Keywords:** click biology, posttranslational modification, protein engineering, protein ligation, protein semisynthesis

## Abstract

Cysteine‐less split inteins have recently emerged as valuable addition to the protein labeling and modification toolbox as they can perform the protein *trans*‐splicing (PTS) reaction under oxidizing conditions. Furthermore, their use is compatible with the chemical labeling and different redox states of cysteines in the extein sequences and hence the protein of interest. However, a rapidly splicing cysteine‐less split intein with one short precursor fragment easily amenable to solid‐phase peptide synthesis was still missing. A chemically synthesized split intein precursor allows for semisynthetic PTS, attractive to introduce fully synthetic sequence segments into the protein of interest. Here, we generate an optimized variant of the highly efficient split CL (cysteine‐less) intein that splices with an ultra‐fast rate on the second scale, comparable to the best performing split inteins. We achieved the nine‐ to 16‐fold increase of the reaction rate by optimizing the artificial split site and the immediately flanking extein residues. Following chemical synthesis of the N‐terminal precursor with an intein fragment of only 26 amino acids, we demonstrated protein semisynthesis by transferring a short fluorescently labeled peptide tag to a protein's N terminus with ultra‐fast kinetics. The optimized split CL intein will thus further expand chemical protein labeling approaches.

## Graphical abstract

Protein *trans*‐splicing (PTS) by split inteins is a powerful approach for protein semisynthesis to generate proteins with site‐specific modifications that are difficult or impossible to introduce biosynthetically. Here, the authors report the first ultra‐fast and cysteine‐less split intein with a sufficiently short N‐terminal fragment amenable to chemical synthesis to carry out semisynthetic PTS independent of redox conditions.

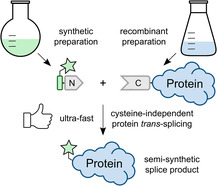

## 1 Introduction

**FIGURE 1:**
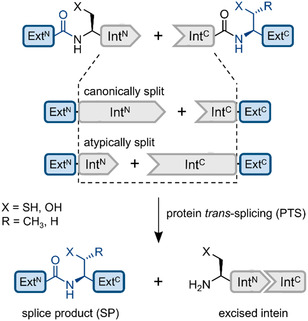
Split intein‐mediated PTS. Split inteins join their flanking sequences, the N‐ and C‐terminal exteins (Ext^N^ and Ext^C^), with a peptide bond. Cysteine‐less inteins operate with Ser1 and Ser + 1/Thr + 1 residues at the two splice junctions. The canonically split inteins comprise an N‐terminal fragment (Int^N^) of about 100−120 aa and a C‐terminal fragment (Int^C^) of about 35−50 aa. This split position with regard to the minimal horseshoe fold of contiguous inteins coincides with the insertion point of a homing endonuclease domain (HEN) found in the so‐called maxi‐inteins. Naturally occurring split inteins with an atypical split site close to the N‐terminal end show short Int^N^ fragments of 15−35 aa.

Selective chemical modification of proteins serves many purposes from biochemical characterizations and cell biology studies to the design of engineered proteins useful in biotechnology or biomedicine, such as protein‐based therapeutics or diagnostics [1, 2, 3]. A large array of protein chemical tools is available to selectively modify and/or substitute amino acid (aa) side chains in proteins [4, 5, 6] or to specifically target fused protein or peptide tags [7]. Other approaches aim to engineer the polypeptide backbone of proteins posttranslationally to introduce or remove (labeled) backbone segments. These include chemical ligation approaches such as native chemical ligation [8, 9] and enzymatic ligation approaches employing subtiligase, sortase, butelase, or other transpeptidases [10, 11, 12, 13, 14]. A unique form of enzymatic ligation can be brought about by split inteins that catalyze protein *trans*‐splicing of two proteins or short peptide tags as single‐turnover catalysts (Figure 1). Split inteins combine the defined and virtually traceless modification of the polypeptide backbone with specific and built‐in high‐affinity recognition of the reaction partners, enabling protein ligation at low concentrations and, for some inteins, with rapid second‐scale kinetics [12, 14, 15].

Protein splicing is an autocatalytic posttranslational reaction wherein the intein as an intervening protein segment undergoes self‐excision out of the protein precursor while concurrently ligating its flanking extein sequences by a native peptide bond [16]. Split intein precursors are expressed as two separate polypeptides with an N‐terminal (Int^N^) and a C‐terminal intein fragment (Int^C^). They associate and fold into the active intein structure to catalyze protein *trans*‐splicing (PTS) (Figure 1) [17, 18, 19]. PTS can occur on the second, minute, or hour‐scale. In particular, ultra‐fast split inteins splicing on the second scale (*t*_1/2_ ≤ 1 min) have emerged as powerful tools in protein engineering and synthetic biology [20, 21, 22, 23].

Protein (*trans*‐)splicing proceeds through a series of coordinated acyl rearrangements of the polypeptide backbone via covalent thioester or ester intermediates (Figure S1) [16]. The intein's first residue (position 1) is a catalytic cysteine or serine residue in the predominant class I inteins [24] and initiates splicing through the formation of the first (thio)ester intermediate. As a part of the intein, this residue is removed from the final spliced protein (Figure 1). In contrast, the first residue of the C‐terminal extein (position +1) is a catalytically essential cysteine, serine, or threonine residue involved in the second (thio)ester intermediate, the branched intermediate (BI), and constitutes the only residue that is strictly required for the reaction to remain as a scar at the ligation site. Most inteins are cysteine‐dependent in their splicing reaction, as they operate at least with a Cys1 residue [25, 26]. This cysteine‐dependency of the split inteins requires that PTS is conducted under reducing conditions as partially oxidized catalytic cysteines (e.g., in form of a disulfide) would lead to a proportional reduction in the splicing yield [26].

Recently, we reported the first cysteine‐less split inteins active at ambient temperature to significantly expand the scope of PTS reaction schemes [23, 26, 27, 28]. Cysteine‐less split inteins are a rare subset of split inteins harboring only serine or threonine residues at the 1 and +1 positions. Ideally, they also lack additional cysteines in their sequence or contain only nonessential cysteines. Importantly, as cysteine‐less inteins do not require reducing agents to achieve full activity, their use is advantageous for proteins that are sensitive to reductive conditions, for example, those containing disulfide bonds [26, 29]. Moreover, in contrast to cysteine‐dependent inteins, they are fully compatible with oxidizing conditions, as present in the extracellular milieu, for example. Furthermore, cysteine‐less split inteins can be combined with thiol bioconjugation chemistries to modify each extein segment independently, thereby providing unique avenues to selective chemical labeling of proteins at one [26, 29, 30], two [23], or even three [28] positions located in the precursor extein segments (three positions by protein assembly from three segments using tandem PTS with two orthogonal cysteine‐less split inteins).

Despite these advances, the toolbox of cysteine‐less split inteins and our knowledge on their biochemical properties is still very limited compared to their cysteine‐dependent counterparts. Only four cysteine‐less split inteins suitable for splicing purified proteins are known, the CLm [23], the LCGC14 [28], the PolB16 OarG [27], and the CL inteins [26]. To fully lift the potential of this family, the identification of more highly active cysteine‐less split inteins is necessary. Only the CLm intein (cysteine‐less and monomeric) belongs to the group of ultra‐fast split inteins that splice within seconds [23]. This intein is derived from the Aes123 PolB1 intein and was rationally mutagenized to prevent Int^N^ precursor aggregation and thereby enable high PTS efficiency [23]. The LCGC14 intein, although splicing virtually quantitatively (≥90%), is much slower with a half‐life time of 8.6 min [28]. Importantly, a cysteine‐less split intein with a short intein fragment and ultra‐fast splicing kinetics is still lacking but would be of particular interest as a protein ligation and click biology [3] tool. Naturally occurring atypically split inteins possess Int^N^ fragments of 15–37 aa [27, 31, 32, 33], substantially shorter than those of canonical split inteins, which typically comprise ca. 100−130 aa (Int^N^) and ca. 35−50 aa (Int^C^) (Figure 1) [19, 34, 35, 36]. Sufficiently short Int^N^ fragments offer several advantages as they minimize potential perturbation of the protein of interest and are conveniently accessible via solid‐phase peptide synthesis (SPPS). Synthetic precursors enable the straight‐forward incorporation of nonnatural chemical modifications in the extein parts, which can then be transferred to the protein of interest in a semisynthetic PTS reaction. The PolB16 intein is the only known naturally occurring, cysteine‐less and atypically split intein. It features a record‐short Int^N^ of only 15 aa with demonstrated utility in semisynthetic PTS [27]. However, its practical value is limited by a rather poor splicing efficiency, including up to 25% of the C‐cleavage side reaction and a rather slow rate of the PTS reaction (*t*_1/2_ > 25 min at pH 7) [27]. Similarly, the CL intein represents an artificially split variant of the parent Aes123 PolB1 intein with a short Int^N^ of only 26 aa [26]. Although it splices with high efficiency (≥90%), it suffers from rather slow splicing rates with a *t*_1/2_ of about 20 min [26]. Such impact on the splicing performance is usually observed for artificially split inteins [17, 36, 37, 38]. Thus, an ultra‐fast cysteine‐less split intein with one short fragment for protein semisynthesis is still sought after.

Furthermore, changes in the residues immediately flanking the intein can impact the splicing performance to widely varying degrees, as it has been studied for several cysteine‐dependent inteins [34, 39, 40, 41, 42, 43, 44]. However, the tolerance of the new cysteine‐less split inteins for nonnative sequence contexts has not been investigated yet.

In this work, we aimed to obtain a cysteine‐less split intein featuring a short Int^N^ fragment with improved activity in PTS and to study its extein residue tolerance. In light of lacking other naturally occurring candidates to test, we revisited the previously reported CL intein with its Int^N^ fragment of 26 aa. Further trimming of the Int^C^ fragment as well as a systematic study of its extein residue preferences surprisingly led to a 16‐fold increase in the PTS rate to the second range. These findings represent a valuable extension of the split intein toolbox for the posttranslational engineering of proteins and offer surprising insights into the factors important for high splicing activity.

## 2 Results and Discussion

### 2.1 An Altered Split Site Deletion in the CL Intein Improves Its Rate in PTS

**FIGURE 2:**
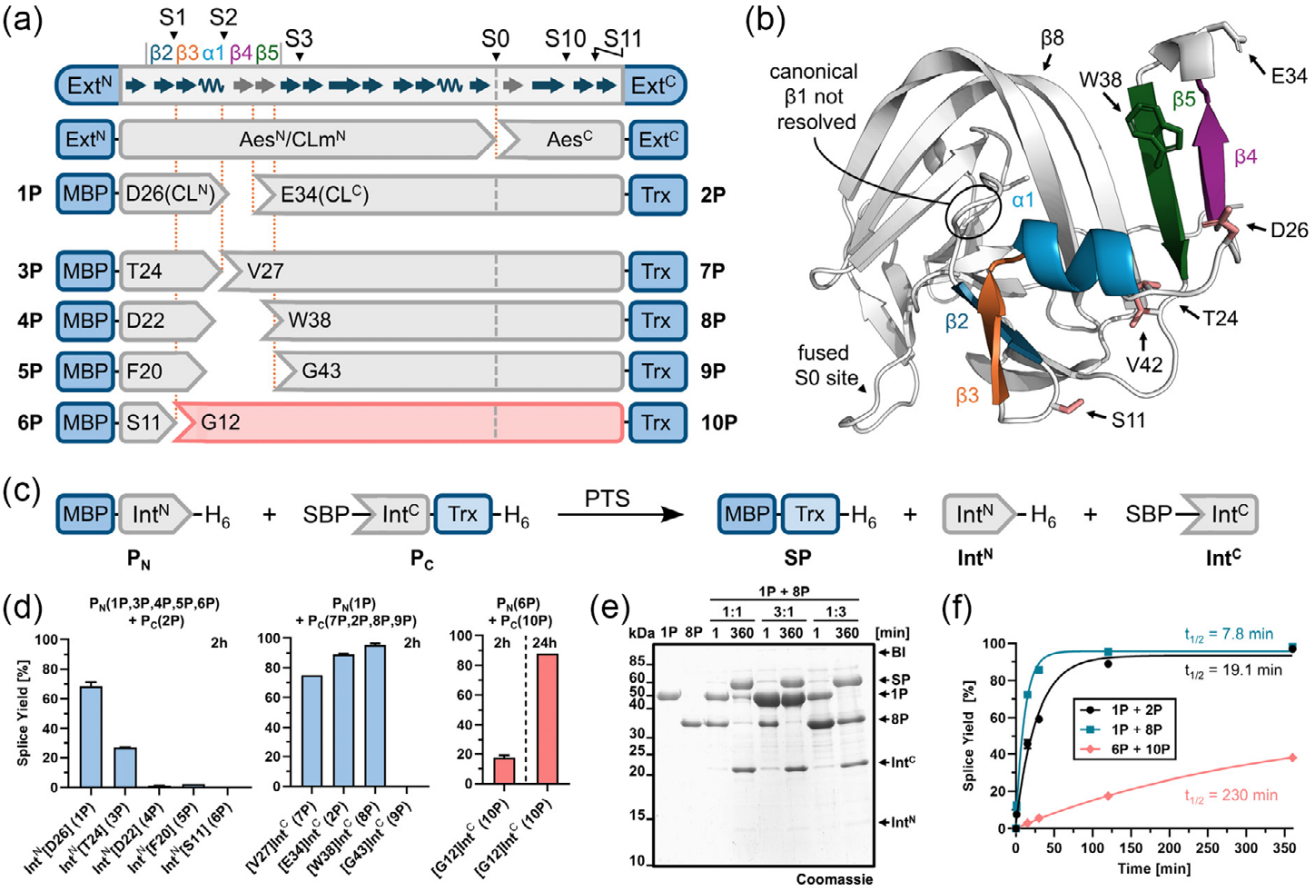
Altered splitsite deletions in the artificially split CL intein. (a) Scheme illustrating the investigated split site deletions in the artificially split CL intein according to conserved secondary structure elements in the minimal intein fold (gray: optional insertions) with the typical (S0) and atypical (S1, S2, S3, S10, S11) split positions numbered according to Sun et al. [37]. Note that the Aes123 PolB1 intein contains an insertion at the S2 site, encompassing β‐strands β4 and β5, relative to the minimal intein structure used for the split position nomenclature. (b) Crystal structure of the Aes123 PolB1 intein (PDB ID: 9HTH), highlighting in color the N‐terminal region from β‐strand β1 to β4, where the split site deletions were introduced. (c) Scheme of the PTS reactions with model proteins to analyze the split site variants. (d) Effect of the Int^N^ truncations on the PTS yield (*P*_*N*_ = 10 µM; *P*_C_ = 10 µM) (left panel; see also Figure S2), effect of the Int^C^ truncations on the PTS yield with one precursor in threefold excess (10 µM and 30 µM) (middle panel; see also Figure S3), and PTS reaction between Int^N^[S11]‐H_6_ (**6P**) and SBP‐[G12]Int^C^ (**10P**) (right panel; see also Figure S4) determined by densitometric SDS‐PAGE analysis. (e) SDS‐PAGE analysis of the PTS reactions to analyze the D26/W38 split site at different molar ratios. (f) Time‐resolved quantification of splice product formation, with data fitted to a one‐phase exponential equation. All reactions were carried out at 37°C and pH 7. Calculated molecular weights are **1P**: 47.3 kDa; **8P**: 33.3 kDa; **SP(1P/8P)**: 57.5 kDa. BI = branched intermediate. Ext = extein. SP = splice product. MBP = maltose binding protein. Trx = thioredoxin. For (d), *n* = 2−3 technical replicates. For (f), *n *= 3 technical replicates. Data are presented as mean ± SD normalized to the molecular weight of the respective protein species.

The previously reported CL intein was generated by artificially splitting the Aes123 PolB1 intein close to the N‐terminal end [26]. The initial split site D26/E34 yielded an Int^N^[D26] fragment of 26 aa and the longer [E34]Int^C^ fragment starting at residue 34 (plus the additional start methionine; Figure 2a). The deletion of residues V27−D33 was chosen at the time based on structural predictions and alignments that indicated that the region of the Aes123 PolB1 intein spanning residues ∼25 and 40 represents an insertion relative to the minimal canonical horseshoe fold of the *Ssp* DnaB mini‐intein and might be superfluous [26]. Here, we aimed to determine whether the CL intein could be improved by optimizing the split position based on the meanwhile published crystal structure of the Aes123 PolB1 intein (PDB ID: 9HTH) [23].

The crystal structure confirmed that the bespoke insertion forms two β‐strands (β4 and β5) that are not found in the minimal horseshoe fold, however, that are commonly observed in cysteine‐independent inteins (Figure 2b) [27]. The previously used split site D26/E34 effectively removes β‐strand β4, indicating it is not essential for folding and splicing. Naturally occurring atypical split sites that generate a short Int^N^ fragment have so far been observed either immediately following β‐strand β2 (also referred to as S1 position [37]), as seen in the cysteine‐dependent VidaL intein [33, 45] and the cysteine‐less PolB16 intein [27], or following the first α‐helix (*α*1) (also referred to as S2 position), as found in the cysteine‐dependent AceL TerL [31] and GOS TerL [32] inteins (Figure 2a,b).

The S2 split site in the Aes123 PolB1 intein would be located after residue T24 (Figure 2b). To explore this position, we shortened the Int^N^ fragment in the reference model construct MBP‐Int^N^[D26]‐H_6_ (**1P**; maltose‐binding protein as the extein) [26] to give MBP‐Int^N^[T24]‐H_6_ (**3P**). Additional truncations were also tested, yielding constructs MBP‐Int^N^[D22]‐H_6_ (**4P**), MBP‐Int^N^[F20]‐H_6_ (**5P**), and MBP‐Int^N^[S11]‐H_6_ (**6P**), the latter corresponding to the S1 site. These proteins were tested for PTS with the C‐terminal reference precursor construct SBP‐[E34]Int^C^‐Trx‐H_6_ (**2P**) [26], which employs thioredoxin (Trx) as model extein and streptavidin‐binding peptide (SBP) as an expression tag (Figure 2c). All precursor proteins were recombinantly expressed in *E. coli* and purified using Ni‐NTA affinity chromatography. Splicing efficiency was evaluated by incubating each N‐terminal precursor with the C‐terminal precursor **2P** at equimolar ratios, in the absence of reducing agents, at 37°C (Figure S2). As summarized in Figure 2d (left panel), SDS‐PAGE analysis revealed a substantial decrease in splicing efficiency for the T24/E34 split site relative to the D26/E34 site and an almost complete loss of activity for the Int^N^ precursors that underwent further shortening. We also tested precursor **6P** (MBP‐Int^N^[S11]‐H_6_) with its S1 site counterpart MBP‐Int^N^[G12]‐H_6_ (**10P**). Interestingly, clean splicing occurred and even could be driven close to completion within 24 h, suggesting potential practical utility. However, because the rate was significantly reduced, we did not explore this variant further in this work (Figures 2a,d,f and S4).

Next, we investigated further deletions on the C‐terminal split intein precursor. To this end, we prepared constructs SBP‐[V27]Int^C^‐Trx‐H_6_ (**7P**), SBP‐[W38]Int^C^‐Trx‐H_6_ (**8P**), and SBP‐[G43]Int^C^‐Trx‐H_6_ (**9P**) and tested all of these in combination with the N‐terminal precursor **1P**. To calculate pseudofirst‐order rate constants, one of the precursors was used in threefold molar excess (Figure S3). Splicing between **1P** and **7P** exhibited reduced efficiency compared to the original D26/E34 split site, with a decrease in the splicing rate from 0.60 ± 0.04 × 10^−3^ s^−1^ to 0.49 ± 0.03 × 10^−3^ s^−1^ (Figure 2d, middle panel; Figure S3f). Interestingly, however, extending the deletion to D26/W38 split site with construct **8P** significantly enhanced the splicing rate by nearly threefold to 1.48 ± 0.07 × 10^−3^ s^−1^, corresponding to a half‐life of 7.8 min (Figures 2d−f and S3). When using either precursor in molar excess over the other, **1P** or **8P**, in both cases, the substoichiometric protein was virtually completely consumed in the reaction (Figure 2e). Finally, the combination of **1P** and **9P** resulted in a complete loss of splicing activity, suggesting that deletions into β‐strand β5 critically affect the intein structure (Figure 2b). The β‐strand β5 is likely important to stabilize β‐strand β8, which participates in the formation of the catalytic center by providing the conserved catalytic block X histidine (His68), a key residue uniquely found in cysteine‐independent inteins [27].

### 2.2 Local Extein Tolerances of the CL Intein at the −1 Position

We investigated the tolerance of the CL intein toward changes in the flanking extein residues. Analysis of the crystal structure of the parental Aes123 PolB1 intein [23] suggested that the potential interaction of most of the extein residues is limited to peripheral regions of the intein with minimal potential effects on splicing (four and six residues at the N‐ and C‐extein, respectively, were included in the crystal structure of the intein inactivated for protein splicing by point mutations of the catalytic Ser1 and Asn159 residues). However, the Asp−1 residue hydrogen‐bonds via its side chain carboxyl group with the *π*‐nitrogen of the catalytic block B/N3 histidine (His90), and thereby adopts an unusual conformation [23]. This interaction may contribute to the distortion of the upstream scissile bond by imposing torsional strain on the peptide bond through the Asp−1 conformation, to help promoting the initial *N−O* acyl shift in addition to other more conserved mechanisms. Thus, the crystal structure suggested that the nature of the −1 side chain, generally considered as the most critical noncatalytic extein residue, may be of particular importance for the splicing activity of the Aes123 PolB1 and CL inteins.

**FIGURE 3:**
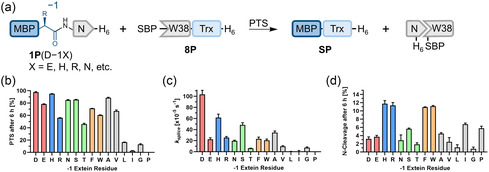
Influence of side chain substitutions at the −1 position on splicing efficiency. (a) Schematic overview of the PTS reactions using various Asp−1 substitutions introduced into the N‐terminal precursor MBP‐Int^N^[D26]‐H_6_ (**1P**). (b–d) Influence of the Asp−1 substitutions on the product yield (b), splicing rate (c), and N‐cleavage levels (d), as determined by time‐resolved densitometric SDS‐PAGE analysis after 6 h, at 37°C and pH 7, with data fitted to a one‐phase exponential equation (see Figure S5 for gels). The color code represents the physicochemical properties of the side chains: acidic (red), basic (blue), polar (green), aromatic (orange), and hydrophobic (gray). For (b–d), *n* = 2 technical replicates. Data are presented as mean ± SD.

We prepared 14 side chain substitutions at the −1 position within the N‐terminal precursor MBP‐Int^N^[D26]‐H_6_ (**1P**), representing all possible side chain physicochemical properties and steric demands. We evaluated their effect by analyzing PTS reactions with the C‐terminal precursor SBP‐[W38]Int^C^‐Trx‐H_6_ (**8P**) used in threefold molar excess (Figure 3a). Notably, most substitutions at the −1 position resulted in acceptable product yields of 50%−90% after 6 h, however, with significant reductions of the splicing rates in most cases (Figure 3b,c).

Interestingly, precursors **1P**(D−1H), **1P**(D−1S), and **1P**(D−1A) exhibited only modest reductions in splicing rate relative to **1P**, despite the distinct physicochemical properties of their −1 side chains. In contrast, precursors **1P**(D−1L), **1P**(D−1I), and **1P**(D−1G) showed an almost complete loss of splicing (Figure 3b,c). The other precursors with *β*‐branched side chains at the −1 position (**1P**(D−1V) and **1P**(D−1T)) still showed decent splicing but with dramatic decreases in the splicing rate (t_1/2_ = 118 min and 188 min, respectively). Proline at the −1 position (**1P**(D−1P)) led to no detectable splicing activity (Figure 3b,c).

These findings indicate that the side chain polarity at the −1 position influences the splicing efficiency of the CL intein, although not in a way predictable by the interaction of the intein with the native D−1 residue as seen in the crystal structure. Bulky amino acid substitutions were frequently associated with elevated N‐cleavage rates of 10%−12%, as observed for precursors **1P**(D−1H), **1P**(D−1R), **1P**(D−1F), and **1P**(D−1W) (Figure 3d). Nevertheless, these substitutions might still be useful for preparative purposes, in addition to the changes D−1S and S−1A.

### 2.3 The CL Intein Efficiently Splices with Both Ser and Thr at the +1 Position

**FIGURE 4:**
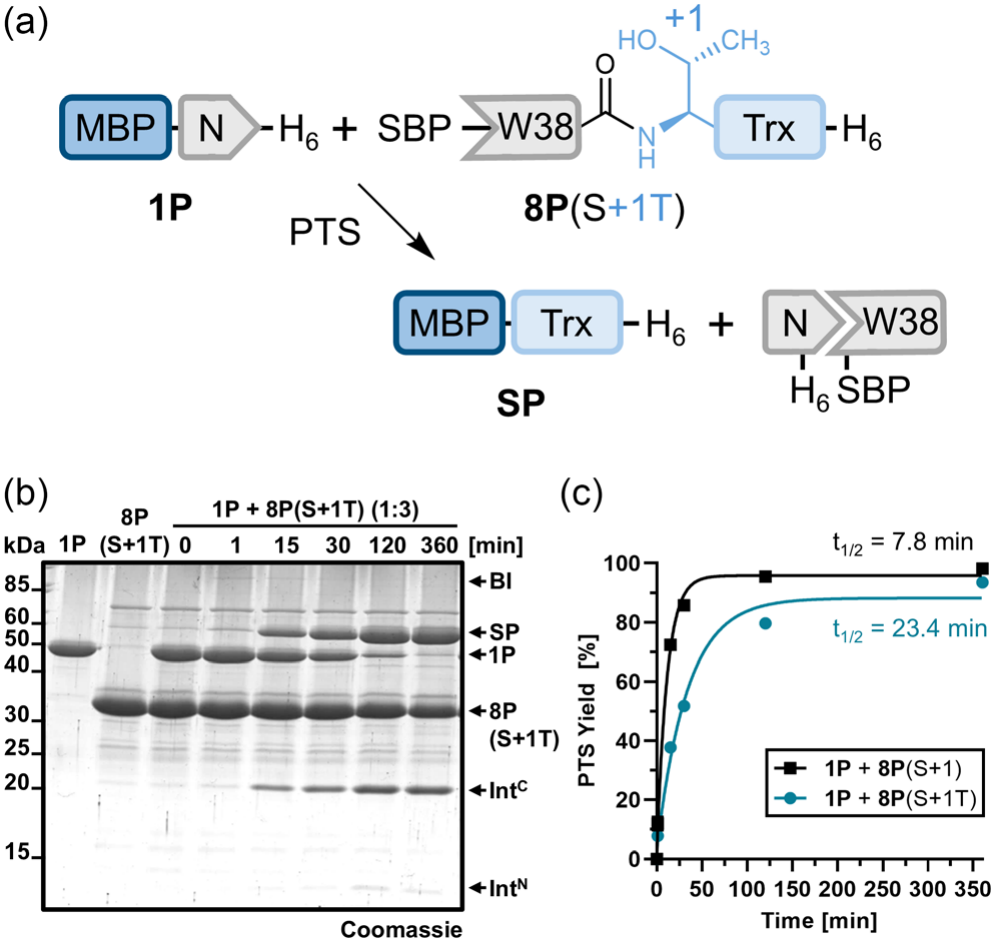
Influence of Ser + 1 substitution with threonine on splicing efficiency. (a) Scheme of the PTS reaction. (b) SDS‐PAGE analysis of the PTS reaction shown in (a) using the C‐terminal precursor in threefold molar excess (10 to 30 µM) at 37°C and pH 7. This experiment was repeated two times. (c) Time‐resolved quantification of splice product formation by densitometric analysis, with data fitted to a one‐phase exponential equation. Calculated molecular weights are **1P**: 47.3 kDa; **8P**(S + 1T): 33.3 kDa; SP: 57.5 kDa. BI = branched intermediate; SP = splice product. For (c), *n* = 2 technical replicates. Data are presented as mean ± SD normalized to the molecular weight of the respective protein species.

We then turned to the +1 extein residue, which also directly participates in catalysis. We previously showed that a S + 1C mutation completely abolished splicing activity despite the higher nucleophilicity of the cysteine side chain [26]. Here, we prepared SBP‐[W38]Int^C^‐(S + 1T)Trx‐H_6_ (**8P**(S + 1T)) and surprisingly observed quantitative splicing with the N‐terminal precursor **1P**, albeit with a threefold reduced rate of 0.49 ± 0.05 × 10^−3^ s^−1^ (*t*_1/2_ = 23.4 min) (Figure 4). The ability of the CL intein to efficiently splice with Thr at the +1 residue, which is the minimal scar of the extein sequences remaining in the splice product, represents a valuable expansion of potential ligation sequences.

### 2.4 Local Extein Tolerances at Other Positions Can Be Exploited for Further Rate Enhancements of the CL Split Intein

**FIGURE 5:**
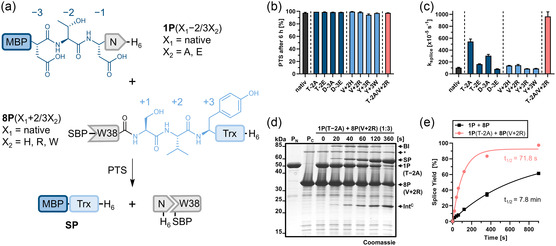
Influence of side chain substitutions at the −2, −3, +2, and +3 positions on splicing efficiency. (a) Schematic overview of the PTS reactions using various local extein substitutions introduced into the N‐terminal precursor MBP‐Int^N^[D26]‐H_6_ (**1P**) and the C‐terminal precursor SBP‐[W38]Int^C^‐Trx‐H_6_ (**8P**), respectively. (b,c) Influence of the side chain substitutions on the product yield (b) and splicing rate (c), as determined by densitometric SDS‐PAGE analysis at 37°C and pH 7 (see Figure S6 for gels). (d) SDS‐PAGE analysis of the PTS reaction using the precursors **1P**(T−2A) and **8P**(V + 2R) in threefold molar excess. This experiment was repeated two times. (e) Time‐resolved quantification of splice product formation by densitometric analysis, with data fitted to a one‐phase exponential equation. Calculated molecular weights are **1P**(T−2A): 47.3 kDa; **8P**(V + 2R): 33.3 kDa; SP: 57.5 kDa. BI = branched intermediate; SP = splice product. For (b, c, e), *n* = 2 technical replicates. Data are presented as mean ± SD. For (e), data are normalized to the molecular weight of the respective protein species.

Next, we examined the tolerance of the CL intein to substitutions at the next layer of residues, i.e., the N‐ and C‐terminal extein positions −2, −3, +2, and +3. Two selected mutations at each of these positions were introduced into each of the constructs MBP‐Int^N^[D26]‐H_6_ (**1P**) and SBP‐[W38]Int^C^‐Trx‐H_6_ (**8P**) within the five native extein residues kept in each precursor (Figure 5a). In line with the structural predictions, we observed no significant impact on the product yield after 6 h of PTS at 37°C, underlining the high extein tolerance of the CL intein (Figure 5b). In some of these cases, the PTS rates were slightly reduced by about 1.2−1.3‐fold compared to the precursors with the native extein context. Surprisingly, however, in other cases, we observed substantial increases in the PTS rates, with the **1P**(T−2A) mutant showing up to a fivefold enhancement (Figure 5c). By combining the fastest N‐terminal precursor **1P**(T−2A) with the fastest C‐terminal precursor **8P**(V + 2R) tested, we could exploit these effects to achieve a splicing rate of 9.7 ± 0.8 × 10^−3 ^s^−1^ (*t*_1/2_ = 72 s; Figure 5d,e), corresponding to a 6.5‐fold increase in the splicing rate compared to the native extein context (with the D26/W38 split site) and to an overall 16‐fold increase relative to the original CL intein (with the D26/E34 split site). The formation of the desired splice product (SP) was confirmed by ESI‐MS analysis (Figure S7). Interestingly, we observed an effect of the extein mutations on a more pronounced accumulation of the branched intermediate (BI) in the course of the PTS reaction (Figures 5d and S8).

### 2.5 The Optimized CL Intein Allows Rapid and Quantitative Semisynthesis of Proteins

**FIGURE 6:**
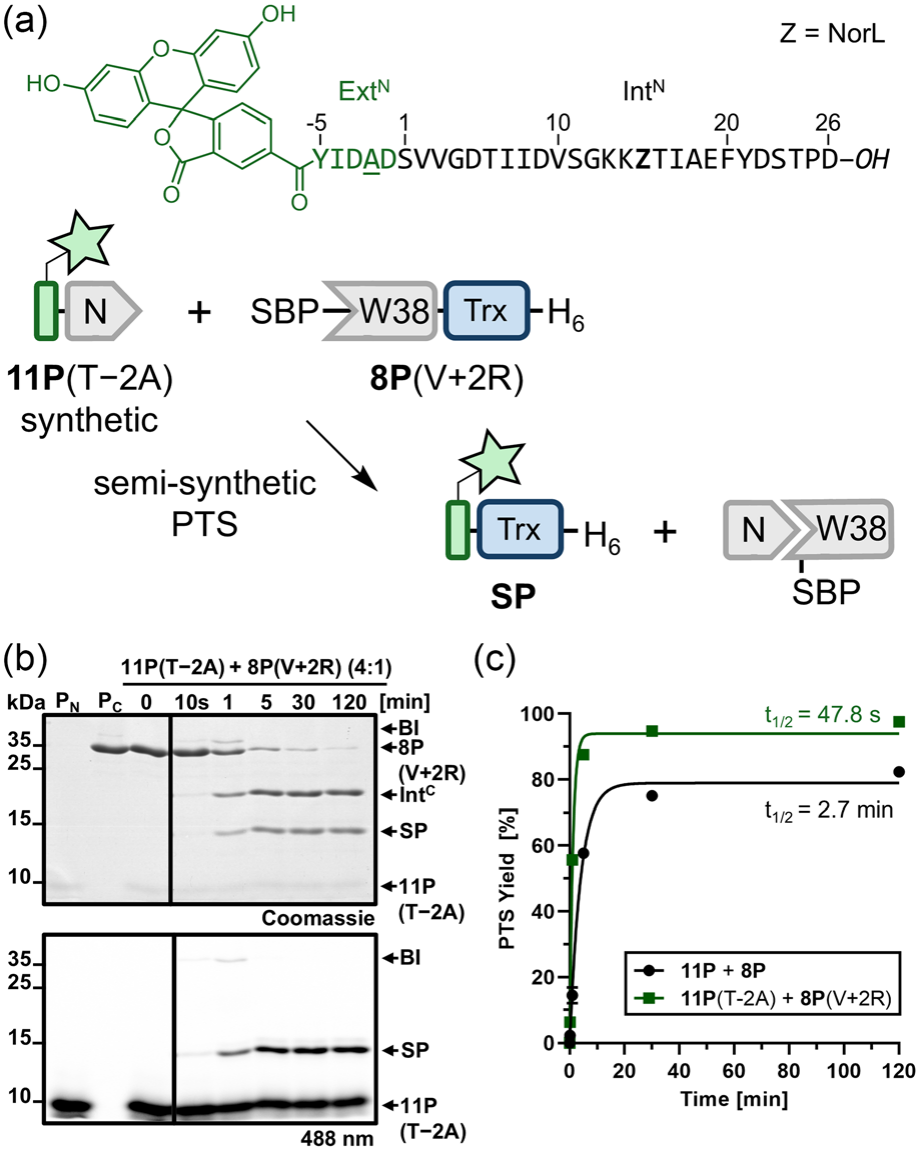
Chemical modification of proteins by semisynthetic PTS using the optimized CL intein. (a) Structure of the synthetic peptide Fl‐Int^N^[D26] with the T−2A substitution (**11P**(T−2A); Fl = 5,6‐carboxyfluorescein) and scheme of the semisynthetic PTS reaction. Note that M15 was replaced with nor‐leucine (Z = NorL) in the synthetic sequence (see also Figure S9). (b) SDS‐PAGE analysis of the PTS reaction followed by fluorescence imaging (lower panel) and Coomassie staining (upper panel) using 40 µM **11P**(T−2A) and 10 µM **8P**(V + 2R) at 37°C and pH 7. This experiment was repeated two times. (c) Time‐resolved quantification of splice product formation by densitometric analysis, with data fitted to a one‐phase exponential equation and compared to data of the PTS reaction with the precursors **11P** and **8P** (see Figure S10). Calculated molecular weights are **11P**(T−2A): 3.7 kDa; **8P**(V + 2R): 33.3 kDa; SP: 15.0 kDa. BI = branched intermediate. SP = splice product. Trx = thioredoxin. Ext = extein. For (c), *n* = 2 technical replicates. Data are presented as mean ± SD normalized to the molecular weight of the respective protein species.

To apply the optimized CL intein variant for chemical modification of proteins with fully synthetic segments, we prepared synthetic Int^N^ precursors by SPPS. The precursor peptide Fl‐Int^N^[D26] (**11P**; Fl = 5,6‐carboxyfluorescein) contained the five native N‐extein residues with a synthetic fluorophore, whereas **11P**(T−2A) featured the accelerating T−2A substitution (Figure 6a). The identity and purity of the synthetic peptides was confirmed by LC‐MS analysis (Figure S9). First, upon incubation of **11P** (40 µM) with the C‐terminal precursor SBP‐[W38]Int^C^‐Trx‐H_6_ (**8P**) (10 µM), we observed quantitative transfer of fluorescein to the Trx extein, as monitored by SDS‐PAGE analysis followed by fluorescence imaging and Coomassie staining (Figures S10). The PTS reaction occurred rapidly with a splicing rate of 4.2 ± 0.3 × 10^−3 ^s^−1^ (*t*_1/2_ = 2.7 min), even about threefold faster than observed for the corresponding fully recombinantly produced split intein (see above). A more rapid PTS reaction when using a synthetic peptide as an N‐precursor is consistent with previous observations for the parent CL intein [26] and might be due to the lacking His_6_‐tag and the smaller N‐extein. We then used **11P**(T−2A) in combination with SBP‐[W38]Int^C^‐Trx‐H_6_ containing the V + 2R accelerating mutation (**8P**(V + 2R)) and observed quantitative splicing with the expected further increase in the splicing rate (14.5 ± 1.3 × 10^−3^ s^−1^; corresponding to *t*_1/2 _= 48 s; Figure 6b,c). The expected SP was confirmed by ESI‐MS analysis (Figure S11). Notably, this indicates a ninefold acceleration in the rate of the semisynthetic PTS reaction compared to the original CL intein and underlines the ultra‐fast reaction kinetics of the optimized CL intein.

### 2.6 The Canonically Split CLm Intein Exhibits Extein Dependence Comparable to the CL Intein

**FIGURE 7:**
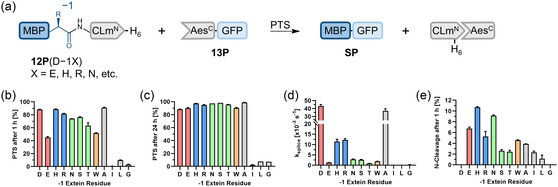
Extein dependence of canonically split CLm/Aes intein. (a) Schematic overview of the PTS reactions. (b–e) Influence of the Asp−1 substitutions on the product yield after 1 h (b), and after 24 h (c), on the splicing rate (d), and on N‐cleavage levels (e), as determined by densitometric SDS‐PAGE analysis at 37°C and pH 7, with data fitted to a one‐phase exponential equation (see Figure S12 for gels). The color code represents the physicochemical properties of the side chains: acidic (red), basic (blue), polar (green), aromatic (orange), and hydrophobic (gray). For (b–e), *n* = 2 technical replicates. Data are presented as mean ± SD.

Finally, we tested whether our insights on extein sequence tolerance of the CL intein could be extended to the CLm intein with its Int^N^ and Int^C^ fragments of 120 and 39 aa [23, 28]. Due to their common sequence origin from the parental Aes123 PolB1 intein, we hypothesized that the CLm and the artificially split CL intein would exhibit comparable extein dependencies. We focused on the tolerance of the CLm intein to −1 substitutions. We selected five substitutions with minor effects in the CL intein (D−1H, D−1R, D−1E, D−1A, D−1S), three with moderate effects (D−1N, D−1W, D−1T), and three with strongly detrimental effects (D−1L, D−1G, D−1I). We introduced these substitutions into the N‐terminal precursor MBP‐CLm^N^‐H_6_ (**12P**) and mixed the purified protein variants with a threefold molar excess of the C‐terminal precursor Aes^C^‐sfGFP (**13P**) (Figure 7a).

Consistent with the observations for the CL intein, CLm precursors **12P**(D−1H), **12P**(D−1R), and **12P**(D−1A) exhibited only modestly affected splicing rates with *t*_1/2 _= 60 s, 57 s, and 19 s, respectively, compared to *t*_1/2_ = 16 s of the CLm precursor **12P** with the native D−1 (Figure 7b,d) [23]. Somewhat deviating from the results with the CL intein, the D−1S mutation affected the splicing rate more significantly in the CLm intein, yet the precursor **12P**(D−1S) still spliced to near completion. Precursors **12P**(D−1L), **12P**(D−1G), and **12P**(D−1I) displayed only negligible SP formation and were thus strongly affected by the mutation, again consistent with the observations for the CL intein. Notably, these were the only tested −1 substitutions for the CLm that failed to reach quantitative splicing even after 24 h (Figures 7c and S12). The correlation in dependence on −1 side chain substitutions between the CL and CLm inteins is significantly demonstrated by a nonparametric Spearman correlation coefficient of *ρ *= 0.87 (*p *= 0.00042) between both data sets. Additionally, the CLm intein exhibited effects from the −1 side chain on slightly increased levels of N‐cleavage side product formation comparable to the observations for the CL intein (Figure 7e), further underlining the similarities between the CLm and CL inteins. The ultra‐fast kinetics and high activity of the CLm intein are further underscored by the finding that even precursor **12P**(D−1T), harboring a difficult β‐branched amino acid at the −1 position, still spliced with a half‐life time of 13 min, which can be acceptable for modifying purified proteins by PTS.

We also tested the CLm intein with the T−2A and V + 2R substitutions that we discovered as optimized extein context of the CL intein. We observed highly efficient and ultra‐fast PTS with an about threefold improved splicing rate (*t*_1/2_  = 6.4 s; Figure S13) compared to the rate of the CLm intein with its native flanking 5 residues on each extein side (*t*_1/2_  = 16.3 s). Yet, when testing SBP as an alternative C‐extein, this rate enhancement was not observed (*t*_1/2_  = 15.4 s; Figure S13). Thus, extein engineering does not lead to a clear rate enhancement in case of the CLm intein. Interestingly, similar to the effect of the extein mutations in the CL intein, we observed a marked accumulation of the branched Intermediate (BI), indicating accelerated kinetics in the formation of the BI, while the succinimide formation remained rate‐limiting within the splice mechanism (Figure S13 and Ref. [23] for the native extein context).

## 3 Conclusion

We report here the optimized CL intein as the first semisynthetic and cysteine‐less split intein that splices with ultra‐fast reaction kinetics (*t*_1/2_ = 48 s to 72 s) and virtually quantitative yields. Notably, the semisynthetic labeling reaction was virtually complete after a few minutes and could be performed in the absence of reducing agents owing to the cysteine‐less nature of the split intein. By using a chemically synthesized Int^N^ precursor that transfers a synthetic fluorophore along with a tag of only 5 amino acids to the protein of interest, we demonstrate the potential of the optimized CL intein for protein semisynthesis. Other ultra‐fast split inteins are the canonically split *Npu* DnaE intein (*t*_1/2_ = 63 s at 37°C) [20, 34], the CLm intein (*t*_1/2_ = 16 s at 37°C) [23], the Gp41‐1, Nrd‐J, and Gp41‐8 inteins (*t*_1/2_ = 5 s, 7 s, and 15 s at 37°C, respectively) [21] and the atypically split VidaL intein (*t*_1/2_ ≈ 1 min at 37°C) [33, 45]. However, except for the CLm intein all these inteins are cysteine‐dependent, and only the VidaL intein has a short Int^N^ fragment. Thus, the ultra‐fast CL intein should represent a unique and valuable addition to the protein labeling toolbox.

In light of the absence of a naturally split, fast, highly efficient, and cysteine‐less atypically split intein with a short intein fragment that is in the range for convenient chemical synthesis by SPPS (≤ca. 25 aa), we revisited the previously reported CL intein. This artificially split intein was obtained by shifting the split site of the natural Aes123 PolB1 intein close to the N‐terminal end [26]. We found that an additional truncation of the CL Int^C^ precursor increased the splicing rate. We then revealed that extein residue alterations at the −2 and +2 positions further enhanced the splicing rate. Both these effects were found to be additive and were observed for splicing reactions with both the fully recombinant split intein precursors as well as the semisynthetic set‐up in which the Int^N^ precursor was a synthetic peptide. When used together, we could show that the optimized CL intein spliced ninefold faster than the previously reported CL intein in case of the semisynthetic reaction set‐up (*t*_1/2_ = 48 s vs. *t*_1/2_ = 10 min, respectively), and even 16‐fold faster in case of the recombinantly produced Int^N^ precursor containing a C‐terminal His_6_‐tag (*t*_1/2_ = 72 s vs. *t*_1/2_ = 20 min, respectively).

With these rate improvements, the artificially split CL intein approaches the rate of the even faster CLm intein (*t*_1/2_ = 16 s). The CLm intein retains the natural split site at the HEN‐position (also referred to as S0 site, Figure 2b), for which the common parent Aes123 PolB1 intein was optimized during the course of evolution. Obviously, the artificial split site of the CL intein variants is the cause for the overall slower PTS rates, as their artificially short Int^N^ and long Int^C^ precursors cannot associate by the native mechanism in the capture‐and‐collapse assembly driven by charge clusters in the native Int^N^ and Int^C^ fragments [23, 46, 47]. This hypothesis is further supported by the lower affinity of the CL^N^ and CL^C^ precursors compared to the Aes123^N^ and Aes123^C^ precursors (*K*_D_ = 1.7 µM vs. 12 nM, respectively) [23, 26]. So why then did the optimized extein context reported herein improve the PTS rate of the CL intein up to sixfold, significantly more than seen in other systematic studies on extein optimization [40, 43]? A telling indication to understand the changes on the molecular level is the significantly more rapid build‐up of the branched intermediate (BI) in conjunction with the extein mutations T−2A and V + 2R that we observed for both the CL and the CLm inteins (Figures 5d, S7 and S12), while the rate of the resolution from the BI to the SP remained unchanged (involving cyclization of intein's block G/C1 asparagine side‐chain as the rate‐determining step) [23, 48]. These findings suggest that following the initial association, the extein‐optimized CL intein much faster undergoes the first steps of the protein splicing mechanism up to the BI and thereby removes the CL^N^ precursor from the initial association equilibrium. The result is that dissociation of the CL^N^ and CL^C^ precursors is reduced, and the once associated CL^N^ and CL^C^ precursors are more likely to progress toward the SP, effectively translating into the higher splicing rate. A similar rate increase of BI formation in the CLm intein, however, did not translate into an overall higher PTS rate for this intein, probably because the initial precursor association equilibrium is already much more in favor of the associated complex and hence does not benefit from the CLm^N^ precursor being more rapidly removed on its way along the first steps of protein splicing. A more detailed biophysical characterization in the future will allow to test this interpretation through determination of the rates of the assembly process.

Together, these results represent a remarkable optimization of PTS rates, in particular with regard to the effect of extein optimization. The changes observed in BI accumulation point at the importance of the coordination and rates of the individual steps along the splicing pathway [48, 49, 50, 51] and at the potential to optimize their fine‐tuning for achieving higher overall rates [51].

One of the restrictions of intein‐mediated ligation is a dependency on the immediately flanking extein residues. We observed a high tolerance for substitutions at the −2/−3 and +2/+3 positions, suggesting that substitutions here and at positions further outside will generally be well tolerated. Consequently, the residual sequence scar at the ligation junction can likely be limited to only 4−6 aa in length, a size comparable to the scars generated by the widely used transpeptidase ligation tools [7]. However, within these 4–6 aa many deviations from the optimal sequence will still result in satisfactory splicing performance, notably also including a change of the catalytic Ser + 1 to Thr + 1. As unsuitable amino acid side chains at the −1 position, we identified Gly, Ile, Leu, and Pro. The highest splicing rate reported here for the CL intein comes at the cost of a ligation scar restricted to at least the 4 residues AD−SR. Such sequence restrictions, however, will not be of concern in many protein labeling applications when a short linker is well tolerated or even intended [29]. Similarly, our insights into the extein dependencies of the CLm intein will also aid in planning reactions with this ultra‐active cysteine‐less split intein variant [23].

The reported split CL intein features a short Int^N^ fragment consisting of 26 aa, conveniently accessible by chemical synthesis using SPPS; a prerequisite for the intended protein semisynthesis. Further truncation resulted in a loss of activity. Notably, other well‐splicing split inteins with short Int^N^ fragments are known and have been applied for semisynthetic PTS, such as the cysteine‐dependent and naturally split Acel TerL (25 aa) [31], TerL CAT (30 aa) [52], VidaL (16 aa) [33, 45], and the artificially split M86 inteins (11 aa) [38]. However, our focus was to establish a semisynthetic split intein tool that is fully cysteine‐independent and hence resistant to oxidative conditions and compatible with thiol chemistry in its extein sequences. The only cysteine‐less split intein with a short Int^N^ fragment is the naturally split PolB16 OarG intein (15 aa), which is of only limited utility due to its slow reaction rate, incomplete splicing efficiency, and high tendency for the C‐terminal cleavage side reaction [27]. A further reduction in length of the Int^N^ fragment of a cysteine‐less split intein to ∼15 aa would be desirable to further simplify the chemical synthesis of N‐terminal precursor peptides. However, it is unclear which inteins can tolerate such a split position even closer to the N‐terminus and how highly efficient and rapid PTS is governed in such cases.

Future studies could be directed at identifying or engineering cysteine‐less split inteins for protein semisynthesis with an even shorter N‐ or C‐terminal fragment. Improvement of rate and extein tolerance has also been successfully achieved for other split inteins by mutational rational engineering and directed evolution of the intein sequence itself [31, 38, 41, 53, 54, 55, 56], providing another option not exhausted in this work. In the interim, the optimized CL split intein reported here should provide a powerful tool for cysteine‐independent chemical protein labeling and semisynthesis.

## Author Contributions

**Christoph Humberg**: conceptualization (equal), data curation (lead), formal analysis (lead), investigation (lead), writing – original draft (equal), writing – review and editing (equal). **Tobias M. E. Terhorst**: formal analysis (supporting), investigation (supporting). **Tim Pasch**: formal analysis (supporting), investigation (supporting). **Henning D. Mootz**: conceptualization (equal), formal analysis (supporting), funding acquisition (lead), project administration (lead), supervision (lead), writing – original draft (equal), writing – review and editing (equal).

## Supporting Information

Additional supporting information can be found online in the Supporting Information section. **Supporting Fig. S1**: Intein protein‐splicing mechanism. General protein‐splicing mechanism of split inteins (X = S, O; R = H, CH_3_). Note that cysteine‐less inteins operate with Ser1 and Ser+1 (Thr+1) residues at the two splice junctions to form the linear and branched ester intermediates. Initially, an N−S/O acyl shift transfers the N‐extein to a Cys or Ser residue at the intein's first position, forming a linear (thio)ester intermediate. In the subsequent trans(thio)esterification step, the Cys, Ser, or Thr side chain from the C‐extein's first position (position +1 with regarding to intein numbering) attacks the linear intermediate to form a branched (thio)ester intermediate. The branched intermediate is then resolved by cyclization of a conserved C‐terminal asparagine, the last residue of the intein, cleaving the bond between the intein and C‐extein, and releasing the intein as a C‐terminal succinimide. Finally, the liberated α‐amino group of the +1 position triggers a spontaneous S/O−N acyl shift, yielding a native peptide bond between the exteins. **Supporting Fig. S2**: Effects of Int^N^ shortening on splicing efficiency of the artificially split CL intein. (a) Scheme of the PTS reactions. (b‐f) SDS‐PAGE analysis of the PTS reactions shown in (a) using the N‐terminal precursors, (b) MBP Int^N^[D26]‐H_6_ (**1P**), (c) MBP‐Int^N^[T24]‐H_6_ (**3P**), (d) MBP‐Int^N^[D22]‐H_6_ (**4P**), (e) MBP‐Int^N^[F20]‐H_6_ (**5P**), and (f) MBP Int^N^[S11]‐H_6_ (**6P**) together with the C‐terminal precursor SBP‐[E34]Int^C^‐Trx‐H_6_ (**2P**) at equimolar concentrations, at 37°C. Shown are Coomassie‐stained gels. These experiments were performed in duplicate. (g) Time‐course of the PTS reactions based on densitometric analysis. BI = branched intermediate; MBP = maltose‐binding protein; SBP = streptavidin‐binding peptide; SP = splice product; Trx = thioredoxin. For (g), *n* = 2−3 technical replicates. Data are presented as mean ± SD normalized to the molecular weight of the respective protein species. **Supporting Fig. S3**: Effects of new split site deletions on the Int^C^ fragment on splicing efficiency of the artificially split CL intein. (a) Scheme of the PTS reactions. (b–e) SDS‐PAGE analysis of the PTS reactions shown in (a) using the N terminal precursors MBP‐Int^N^[D26]‐H_6_ (**1P**) together with the C‐terminal precursors (b) SBP‐[V27]Int^C^‐Trx‐H_6_ (7P), (c) SBP‐[E34]Int^C^‐Trx‐H_6_ (**2P**), (d) SBP‐[W38]Int^C^‐Trx‐H_6_ (**8P**), and (e) SBP‐[G43]Int^C^‐Trx‐H_6_ (**9P**) with one of the precursors given in threefold molar excess as indicated, at 37°C, shown are Coomassie‐stained gels. These experiments were performed in duplicate or triplicate. (g) Time‐resolved quantification of splice product formation by densitometric analysis, with data fitted to a one‐phase exponential equation. BI = branched intermediate; MBP = maltose‐binding protein; SBP = streptavidin‐binding peptide; SP = splice product; Trx = thioredoxin. For (f), *n* = 2−3 technical replicates. Data are presented as mean ± SD normalized to the molecular weight of the respective protein species. **Supporting Fig. S4**: Efficiency of the S11/G12 split site. (a) Scheme of the PTS reaction. (b,c) SDS‐PAGE analysis of the PTS reaction shown in (a) using the N‐terminal precursors MBP‐Int^N^[S11]‐H_6_ (**6P**) together with the C‐terminal precursors SBP‐[G12]Int^C^‐Trx‐H_6_ (**10P**) with one of the precursors given in three‐ or fourfold molar excess as indicated, at 37°C. Shown are Coomassie‐stained gels. The experiment in (c) was performed in triplicate. (d) Quantification of splice product formation after 24 h by densitometric analysis of the data shown in (b). MBP = maltose‐binding protein; SBP = streptavidin‐binding peptide; SP = splice product; Trx = thioredoxin. **Supporting Fig. S5**: Analysis of the D−1X substitutions introduced into precursor MBP‐Int^N^[D26]‐H_6_ (**1P**) (additional data to Figure 2). Coomassie‐stained SDS‐PAGE analysis of the PTS reactions using the D−1X substitutions introduced into the N‐terminal precursor **1P** with the C‐terminal precursor SBP‐[W38]Int^C^‐Trx‐H_6_ (**8P**) used in threefold molar excess at 37°C. These experiments were performed in duplicate. BI = branched intermediate; SP = splice product. **Supporting Fig. S6**: Analysis of the side chain substitutions at the −2, −3, +2, and +3 positions (additional data to Figure 4). Coomassie‐stained SDS‐PAGE analysis of the PTS reactions using the substitutions introduced into the N‐terminal precursor MBP‐Int^N^[D26]‐H_6_ (**1P**) or the C‐terminal precursor SBP‐[W38]Int^C^‐Trx‐H_6_ (**8P**), respectively. The C terminal precursor is used in threefold molar excess at 37°C. These experiments were performed in duplicate. BI = branched intermediate; SP = splice product. **Supporting Fig. S7**: LC‐MS analysis of the PTS reaction using 1P(T−2A) and **8P**(V+2R). Additional data to the PTS reaction analyzed in Figure 5d,e. (a) RP‐HPLC analysis on a C3 column (ZORBAX StableBond 300 C3, 4: 6 x 12:5 mm, 5 µm, Agilent) of the total PTS reaction using **1P**(T−2A) (5 µM) and **8P**(V+2R) (15 µM) at 37°C. Shown are the UV chromatograms at 280 nm before PTS (black) and 1 h after PTS induction (green). (b) Deconvoluted masses of the ESI‐MS analysis after HPLC separation (7.0–10.0 min) as shown in (a). SP = splice product. **Supporting Fig. S8**: Analysis of the T−2A/V+2R double extein mutation in the CL intein. (a) Scheme of the PTS reaction. (b) SDS‐PAGE analysis of the PTS reaction using the N‐terminal precursors **1P** with the C‐terminal precursors **8P** used in threefold molar excess at 37°C. This experiment was repeated two times. Shown is an exemplary Coomassie‐stained gel. (c) Time‐resolved quantification of splice product (SP) and branched intermediate (BI) formation relative to the precursor **1P** by densitometric analysis, with data fitted to a simplified three‐state kinetic model. (d) Time‐resolved quantification of SP and BI formation relative to the precursor **1P**(T−2A) by densitometric analysis, with data fitted to a simplified three‐state kinetic model (see Method section). BI = branched intermediate; MBP = maltose‐binding protein; SBP = streptavidin‐binding peptide; SP = splice product; Trx = thioredoxin. For (c,d), *n* = 2 technical replicates. Data are presented as mean ± SD normalized to the molecular weight of the respective protein species. **Supporting Fig. S9**: LC‐MS analysis of the synthetic peptides **11P** and **11P**(T−2A). (a) ESI‐MS analysis of the purified synthetic peptide **11P** with M(obs.) 3735.25 Da and M(calc.) 3736.69 Da. (b) ESI‐MS analysis of the purified synthetic peptide **11P**(T−2A) with M(obs.) 3706.18 Da and M(calc.) 3706.67 Da. (c/d) RP‐HPLC analysis of the purified synthetic peptides **11P** (c) and **11P**(T−2A) (d) monitored at 280 nm. **Supporting Fig. S10**: Chemical modification of proteins by semisynthetic PTS using the optimized CL intein (additional data to Figure 6). (a) Sequence of the fluorescein‐labeled synthetic peptide Fl‐Int^N^[D26] (**11P**) and scheme of the semi synthetic PTS reaction with the C‐terminal precursor **8P**. Note that M15 was replaced with nor‐leucine (Z = NorL) in the synthetic sequence. (b/c) SDS‐PAGE analysis of the PTS reaction followed by and Coomassie staining (b) and fluorescence imaging (c) using 40 µM **11P** and 10 µM **8P** at 37°C. This experiment was performed in duplicate. BI = branched intermediate; SP = splice product; Trx = thioredoxin; Ext = extein. **Supporting Fig. S11**: LC‐MS analysis of the splice products of the PTS reactions using the synthetic peptides **11P** and **11P**(T−2A). Additional data to the PTS reactions analyzed in Figure 6 and Figure S8. (a) MS spectrum of the splice product (SP) generated with between 11P (40 µM) and 8P (10 µM) at 37 °C. (b) MS spectrum of the splice product (**SP**) generated with between **11P**(T−2A) (40 µM) and **8P**(R+2V) (10 µM) at 37 °C. (c,d) Deconvoluted masses of the ESI‐MS analysis shown in (a) and (b), respectively. SP = splice product. **Supporting Fig. S12**: Analysis of the D−1X substitutions introduced into precursor MBP‐CLmN‐H6 (**12P**) (additional data to Figure 6). Shown are exemplary Coomassie‐stained SDS‐PAGE gels to analyze the PTS reactions of the N‐terminal precursor **12P** with the indicated D−1X substitutions together with the C‐terminal precursor AesC‐sfGFP (**13P**) used in threefold molar excess at 37°C. This experiment was performed in duplicate. **Supporting Fig. S13**: Analysis of the T−2A/V+2R double extein mutation in the CLm intein. (a) Scheme of the PTS reaction. (b) SDS‐PAGE analysis of the PTS reaction shown in (a) using the N‐terminal precursors **12P**(T−2A) with the C terminal precursors **13P**(V+2R) used in threefold molar excess at 37°C. Shown is an exemplary Coomassie‐stained gel. (c) Time‐resolved quantification of splice product (SP) and branched intermediate (BI) formation relative to the precursor **12P**(T−2A) by densitometric analysis, with data fitted to a simplified three‐state kinetic model (see Method section). (d) Scheme of the PTS reaction. (e) SDS‐PAGE analysis of the PTS reaction shown in (d) using **12P**(T−2A) with the C‐terminal precursors **14P**(V+2R) used in threefold molar excess at 37°C. Shown is an exemplary Coomassie‐stained gel. (f) Time‐resolved quantification of splice product (SP) and branched intermediate (BI) formation relative to the precursor **12P**(T−2A) by densitometric analysis, with data fitted to a simplified three‐state kinetic model. MBP = maltose‐binding protein. SBP = streptavidin‐binding‐peptide. For (c, f), *n* = 2 technical replicates. Data are presented as mean ± SD normalized to the molecular weight of the respective protein species. **Supporting Fig. S14**: Unprocessed SDS‐PAGE images of the figures shown in the main text. The black frame indicates the section used for the figures (a) Figure 2e, (b) Figure 4b, (c) Figure 5d, (d) Figure 6b (upper panel), (e) Figure 6b (lower panel). PageRuler unstained protein ladder (Thermo Scientific #26614) or PageRuler prestained protein ladder (Thermo Scientific #26616) were used as marker. **Supporting Fig. S15**: Unprocessed SDS‐PAGE images of the figures shown in the supplementary information. The black frame indicates the section used for the figures (a) Figure S2b, (b) Figure S2c, (c) Figure S2d, (d) Figure S2e, (e) Figure S2f, (f) Figure S3b, (g) Figure S3c, (h) Figure S3d, (i) Figure S3e, (j) Figure S7b, (k) Figure S9b, (l) Figure S9c, (m) Figure S12b. PageRuler unstained protein ladder (Thermo Scientific #26614) or PageRuler prestained protein ladder (Thermo Scientific #26616) were used as marker. **Supporting Table S1:** List of purified recombinant protein constructs and their expression plasmids. **Supporting Table 2:** List of sequences of recombinantly produced proteins.

## Funding

This study was supported by the Deutsche Forschungsgemeinschaft (DFG) (Grant MO1073/9‐1).

## Conflicts of Interest

The authors declare no conflicts of Interest.

## Supporting information

Supplementary Material

## Data Availability

The data that support the findings of this study are available from the corresponding author upon reasonable request.
