# Supplementary material for: Generating Ultra‐Fast Protein trans‐Splicing of a Cysteine‐Less and Semisynthetic Split Intein for Chemical Protein Labeling
Source: Chembiochem. 2026 Mar 24;27(6):e202500969. doi: 10.1002/cbic.202500969 (PMC13014065; doi:10.1002/cbic.202500969)
Supplement: Supplementary file 1 — Supplementary Material [file CBIC-27-e202500969-s001.pdf]

Supplementary Information

for

Generating Ultra-Fast Protein *trans*-Splicing of a Cysteine-Less and Semi-Synthetic Split Intein for Chemical Protein Labeling

Christoph Humberg,[1] Tobias M. E. Terhorst,[1] Tim Pasch, [1] and Henning D. Mootz[1]*

[1]Institute of Biochemistry, University of Münster, Correnstraße 36, 48149 Münster, Germany

*corresponding author: Henning.Mootz@uni-muenster.de

Table of Contents Page

Experimental Section

Heterologous production of recombinant proteins and purification **S2**

Protein *trans*-splicing assay **S2**

Densitometric analysis and determination of protein *trans*-splicing rate constants **S2**

Solid phase peptide synthesis **S4**

Analytical RP-HPLC and ESI-MS analysis of peptides **S4**

ESI-MS analysis of intact proteins **S4**

Statistical analysis **S5**

Supplementary Tables and Figures

**Table S1**	List of purified recombinant protein constructs and their expression plasmids.	**S6**
**Table S2**	List of sequences of recombinantly produced proteins.	**S7**
**Figure S1**	Intein protein splicing mechanism.	**S8**
**Figure S2**	Effects of Int^N shortening on splicing efficiency of the artificially split CL intein.	**S9**
**Figure S3**	Effects of new split site deletions on the Int^C fragment on splicing efficiency of the artificially split CL intein.	**S10**
**Figure S4**	Efficiency of the S11/G12 split site	**S10**
**Figure S5**	Analysis of the D−1X substitutions introduced into precursor MBP-Int^N[D26]-H_6.	**S11**
**Figure S6**	Analysis of the side chain substitutions at the −2, −3, +2, and +3 positions.	**S12**
**Figure S7**	LC-MS analysis of the PTS reaction using 1P(T−2A) and 8P(V+2R).	**S12**
**Figure S8**	Analysis of the T−2A/V+2R double extein mutation in the CL intein.	**S13**
**Figure S9**	LC-MS analysis of the synthetic peptides 10P and 10P(T−2A).	**S13**
**Figure S10**	Chemical modification of proteins by semi-synthetic PTS using the optimized CL intein	**S14**
**Figure S11**	LC-MS analysis of the PTS reaction using the synthetic peptides 10P and 10P(T−2A).	**S14**
**Figure S12**	Analysis of the D−1X substitutions introduced into precursor MBP-CLm^N-H_6.	**S15**
**Figure S13**	Analysis of the T−2A/V+2R double extein mutation in the CLm intein.	**S16**
**Figure S14**	Unprocessed SDS-PAGE images of the figures shown in the main text.	**S16**
**Figure S15**	Unprocessed SDS-PAGE images of the figures shown in the supplementary information	**S17**

References **S18**

Experimental Section

Heterologous production of recombinant proteins and purification

The overexpression of the protein encoding genes was performed in *E. coli* LOBSTR-BL21 (DE3) or *E. coli* SHuffle® T7 cells. The cells were grown in LB medium at 37 °C or 30 °C, respectively, until an optical density at 600 nm between 0.6 and 0.8 was reached. Expression was induced at 28 °C for 4 h or at 18 °C for 16 h by addition of either 0.4 mM IPTG or 0.2% (w/v) L-arabinose, depending on the plasmid. Cells were harvested by centrifugation (JLA-9100, 4500 rpm for 15 min at 4 °C) and ruptured either using an Avestin EmulsiFlex®-C5 homogenizer (Mannheim, D) or by pulse sonication (10 s on/ 15 s off).

Proteins were purified by Ni-NTA affinity chromatography at 4 °C using gravity flow columns (Cube Biotech) in Ni-NTA buffer (50 mM Tris, 300 mM NaCl, 20 mM imidazole, pH 8.0), washed with increasing amounts of imidazole (20 mM to 40 mM), and then eluted with the same buffer containing 250 mM imidazole.

Proteins bearing an N-terminal H_6-Smt3 tag were purified using a Ni-NTA gravity-flow column as described above. After further purification by semi-preparative size-exclusion chromatography (SEC; Superdex200, GE Healthcare) the eluted protein was treated with 500 nM His_6-tagged Ulp1 for 30 min at 8 °C. After passing the protein mixture over Ni-NTA resin to remove Ulp1 and the cleaved Smt3 tag, the desired product was obtained in the flowthrough.

Protein concentrations were determined using the calculated extinction coefficients at 280 nm. The identity of the products was confirmed by ESI-MS and the purity was assessed by SDS-PAGE or analytical RP-HPLC.

Protein *trans*-splicing assay

Reactions were started by mixing the N- and C-terminal intein precursor proteins in SP buffer (50 mM Tris, 300 mM NaCl, 1 mM EDTA, pH 7.0) at indicated concentrations at 37 °C and in absence of reducing agents. At indicated time points aliquots were removed and the reaction was stopped by adding 4x SDS-PAGE loading buffer (500 mM Tris/HCl, 8% (w/v) SDS, 40% (v/v) glycerine, 20% (v/v) β-mercaptoethanol, 5 mg/L bromophenol blue, pH 6.8) and boiling (95 °C, 5 min). Splice product formation was analyzed by SDS-PAGE. The protein *trans*-splicing assays were at least performed in duplicate.

Densitometric analysis and determination of protein *trans*-splicing rate constants

Coomassie-stained bands were analyzed using Gel Analyzer (v2010a) and normalized to the corresponding molecular weight. Normalized intensities were used to calculate the ratio x of

splice product to the precursor protein used in limiting amount to determine the splice yield as follows.

$$P(\%) = \frac{(100 \times x)}{100 + x} \tag{1}$$

To determine the splice rate the splice product formation was treated as a pseudo-first-order reaction with one of the precursors given in three-fold molar excess and plotted against time. The following single exponential function was fitted to the data using GraphPad Prism (v8). The standard mean derivation is represented by error bars.

$$[P]_t = P_{max}(1 - e^{-k_{total}t}) \tag{2}$$

where P is the normalized intensity of the splice product and k_{total} describes the pseudo-first-order rate equation of the protein trans-splice reaction. The variable t is the reaction time in seconds and P_{max} is a normalization factor which represents the fraction of active precursor protein.

In order to fit a simplified three-state kinetic model as described by Shah *et al.*[1] to the experimental data, the normalized intensities of the precursor protein which was used in limiting amount [A], the normalized intensity of the branched intermediate [BI] and the normalized intensity of the splice product [P] were used. The global fit was based on a system of equations which are the analytical solution to the coupled differential rate equations for those species and carried out using GraphPad Prism (v8).

$$\begin{aligned}
p &= k_1 + k_2 + k_3 \\
q &= \sqrt{p^2 - 4(k_1 k_3} \\
a &= \frac{1}{2}(p + q) \\
b &= \frac{1}{2}(p - q) \\
[A]_t &= P_{max}\left[\left(\frac{k_1(a - k_3)}{a(a-b)}\right)e^{-at} + \left(\frac{k_1(k_3 - b)}{b(a-b)}\right)e^{-bt}\right] \\
[BI]_t &= P_{max}\left[\left(\frac{-k_1 a}{a(a-b)}\right)e^{-at} + \left(\frac{k_1 b}{b(a-b)}\right)e^{-bt}\right] \\
[P]_t &= P_{max}\left[\left(\frac{k_1 k_3}{ab}\right) + \left(\frac{k_1 k_3}{a(a-b)}\right)e^{-at} - \left(\frac{k_1 k_3}{b(a-b)}\right)e^{-bt}\right]
\end{aligned} \tag{3}$$

In these equations, p, q, a, and b are algebraic combinations of rate constants k_1, k_2, and k_3. P_{max} is analogous to the normalization factor described above.

Solid phase peptide synthesis

Peptides were synthesized by microwave-assisted Fmoc-SPPS on Wang resin using a Liberty peptide synthesizer (0.1 mmol scale) as previously described.[2] Fmoc-protected amino acids (Merck Millipore or Iris Biotech) were coupled using Oxyma/DIC activation (1:1) in DMF (4 eq. relative to resin loading), with coupling performed for 45 min at 35 °C, 15 min at 50 °C, or 5 min at 70 °C. After each coupling, capping was carried out with 0.3 M acetic anhydride/ 0.3 M iPr_2NEt in DMF, followed by Fmoc deprotection using 20% piperidine in DMF for 10 min at room temperature (rt). Resin washing with DMF was performed between all steps. Fluorescein labeling was achieved by manual coupling of 5(6)-carboxyfluorescein (1.5 eq.) (Sigma Aldrich) activated with DIC and HOAt (1.5 eq. each) in DMF for 16 h at rt. After washing with DMF and DCM, peptides were cleaved from the resin using ice-cold TFA/TIS/H_2O (90:5:5, v/v/v) for 2 h at rt and purified by RP-HPLC.

Analytical RP-HPLC and ESI-MS analysis of synthetic peptides

RP-HPLC analysis was carried out using an Agilent 1260 Infinity series system (Agilent Technologies) with a multiple wavelength detector SL and a single quadrupole mass spectrometer (Agilent). Samples were diluted with 95% H_2O, 5% acetonitrile and 0.1% TFA and centrifuged (20,000 x g, 2 min). According to the sample concentration, an appropriate volume was loaded on an analytical C4 column (Advance Bio RP-mAb C4, 2.1 mm x 50 mm, 3.5 µm, Agilent) at a flow rate of 0.6 mL/min. Proteins were separated by gradual elution with 5−95% buffer B in 5 min (eluent A: 0.1% formic acid in water; eluent B: 0.1% formic acid in acetonitrile) followed by a washing step with 95% buffer B for 3 min. Absorbance was recorded at 280 nm.

ESI-MS analysis of intact proteins

Mass analysis of intact proteins was performed using an UltiMate™ 3000 RS system (Thermo Fisher Scientific GmbH) connected to a maXis II UHR-qTOF mass spectrometer (Bruker Daltonik GmbH) with a standard ESI source (Apollo, Bruker Daltonik GmbH). Samples were acidified using a 10% formic acid solution to reach a pH 2-3 and centrifuged (20,000 x g, 3 min). According to the protein concentration, an appropriate volume of the supernatant was loaded on a C3 column (ZORBAX StableBond 300 C3, 4.6 x 12.5 mm, 5 µm, Agilent) at a flow rate of 0.3 mL/min in 5% eluent B (eluent A: 0.1% formic acid in water; eluent B: 0.1% formic acid in acetonitrile). After a desalting period of 2 min at 5% B, a gradient was applied (5-95% B in 6 min). MS settings: capillary voltage 4500 V, endplate offset 500 V, nebulizer 3.5 bar, dry gas 8.0 L/min, dry T = 200 °C, mass range m/z 300-3000. Data were analyzed with DataAnalysis (v5.3) (Bruker Daltonik GmbH) and deconvolution was performed using the MaxEnt algorithm implemented in the software.

Statistical analysis

Data are presented as mean ± standard deviation (SD) normalized to the molecular weight of the respective protein species. No statistical method was used to predetermine the sample size. However, we conducted relevant experiments with sample sizes sufficient to determine statistically significant differences across samples. The sample size (*n*) for each analysis is indicated in the respective figure legends. No data were excluded from the analyses. *p*-values are derived from a two-tailed non-parametric Spearman correlation test. All statistical tests on experimental groups were performed using GraphPad Prism software (v8).

Supplementary Tables

Table S1 List of purified recombinant protein constructs and their expression plasmids.

Protein	Construct	Encoding Plasmid	Vector System	Reference
**1P**	MBP-Int^N[D26]-H_6	pTT32	pMAL-c2x	Bhagawati *et al.*[3]
**1P**(D−1E)	MBP-(D−1E)Int^N[D26]-H_6	pCH07	pMAL-c2x	this work
**1P**(D−1H)	MBP-(D−1H)Int^N[D26]-H_6	pCH67	pMAL-c2x	this work
**1P**(D−1R)	MBP-(D−1R)Int^N[D26]-H_6	pCH66	pMAL-c2x	this work
**1P**(D−1N)	MBP-(D−1N)Int^N[D26]-H_6	pCH34	pMAL-c2x	this work
**1P**(D−1S)	MBP-(D−1S)Int^N[D26]-H_6	pTT102	pMAL-c2x	this work
**1P**(D−1T)	MBP-(D−1T)Int^N[D26]-H_6	pCH35	pMAL-c2x	this work
**1P**(D−1F)	MBP-(D−1F)Int^N[D26]-H_6	pCH42	pMAL-c2x	this work
**1P**(D−1W)	MBP-(D−1W)Int^N[D26]-H_6	pCH74	pMAL-c2x	this work
**1P**(D−1A)	MBP-(D−1A)Int^N[D26]-H_6	pTT94	pMAL-c2x	this work
**1P**(D−1V)	MBP-(D−1V)Int^N[D26]-H_6	pCH06	pMAL-c2x	this work
**1P**(D−1L)	MBP-(D−1L)Int^N[D26]-H_6	pCH65	pMAL-c2x	this work
**1P**(D−1I)	MBP-(D−1I)Int^N[D26]-H_6	pCH72	pMAL-c2x	this work
**1P**(D−1G)	MBP-(D−1G)Int^N[D26]-H_6	pTT101	pMAL-c2x	this work
**1P**(D−1P)	MBP-(D−1P)Int^N[D26]-H_6	pCH73	pMAL-c2x	this work
**1P**(T−2A)	MBP-(T−2A)Int^N[D26]-H_6	pCH08	pMAL-c2x	this work
**1P**(T−2E)	MBP-(T−2E)Int^N[D26]-H_6	pCH09	pMAL-c2x	this work
**1P**(D−3A)	MBP-(D−3A)Int^N[D26]-H_6	pCH10	pMAL-c2x	this work
**1P**(D−3E)	MBP-(D−3E)Int^N[D26]-H_6	pCH11	pMAL-c2x	this work
**2P**	SBP-[E34]Int^C-Trx-H_6	pTT43	pET16b	Bhagawati *et al.*[3]
**3P**	MBP-Int^N[T24]-H_6	pTT98	pMAL-c2x	this work
**4P**	MBP-Int^N[D22]-H_6	pTT99	pMAL-c2x	this work
**5P**	MBP-Int^N[F20]-H_6	pTT100	pMAL-c2x	this work
**6P**	MBP-Int^N[S11]-H_6	pTT31	pMAL-c2x	this work
**7P**	SBP-[V27]Int^C-Trx-H_6	pTT104	pET16b	this work
**8P**	SBP-[W38]Int^C-Trx-H_6	pTT105	pET16b	this work
**8P**(S+1T)	SBP-[W38]Int^C(S+1T)-Trx-H_6	pCH12	pET16b	this work
**8P**(V+2H)	SBP-[W38]Int^C(V+2H)-Trx-H_6	pCH13	pET16b	this work
**8P**(V+2R)	SBP-[W38]Int^C(V+2R)-Trx-H_6	pCH14	pET16b	this work
**8P**(Y+3R)	SBP-[W38]Int^C(Y+3R)-Trx-H_6	pCH15	pET16b	this work
**8P**(Y+3W)	SBP-[W38]Int^C(Y+3W)-Trx-H_6	pCH16	pET16b	this work
**9P**	SBP-[G43]Int^C-Trx-H_6	pCH99	pET16b	this work
**10P**	SBP-[G12]Int^C-Trx-H_6	pTT51	pET16b	this work
**11P**	Fl-YIDTD-Int^N[D26]	pepTP03	-	Bhagawati *et al.*[3]
**11P**(T−2A)	Fl-YIDAD-Int^N[D26]	pepTP20	-	this work
**12P**	MBP-CLm^N-H_6	pCH145	pMAL-c2x	Humberg *et al.*[4]
**12P**(D−1E)	MBP-(D−1E)CLm^N-H_6	pCH253	pMAL-c2x	this work
**12P**(D−1H)	MBP-(D−1H)CLm^N-H_6	pCH254	pMAL-c2x	this work
**12P**(D−1R)	MBP-(D−1R)CLm^N-H_6	pCH255	pMAL-c2x	this work
**12P**(D−1N)	MBP-(D−1N)CLm^N-H_6	pCH252	pMAL-c2x	this work
**12P**(D−1T)	MBP-(D−1T)CLm^N-H_6	pCH257	pMAL-c2x	this work
**12P**(D−1W)	MBP-(D−1W)CLm^N-H_6	pCH256	pMAL-c2x	this work
**12P**(D−1I)	MBP-(D−1I)CLm^N-H_6	pCH251	pMAL-c2x	this work
**12P**(D−1L)	MBP-(D−1L)CLm^N-H_6	pCH249	pMAL-c2x	this work
**12P**(D−1G)	MBP-(D−1G)CLm^N-H_6	pCH250	pMAL-c2x	this work
**13P**	Aes^C-sfGFP<br>*precursor*: H_6-Smt3-Aes^C-sfGFP	pCH196	pET28b	Humberg *et al.*[4]
**14P**(V+2R)	SBP-Aes^C(V+2R)-SBP	pCH50	pET16b	this work

Table S2 List of sequences of recombinantly produced proteins.*

Protein	Construct	Sequence
**1P**	MBP-Int^{N}[D26]-H$_6$	MKTEEGKLVIWINGDKGYNGLAEVGKKFEKDTGIKVTVEHPDKLEEKFPQVAATGDGPDIIFWAHDRFG<br>GYAQSGLLAEITPDKAFQDKLYPFTWDAVRYNGKLIAYPIAVEALSLIYNKDLLPNPPKTWEEIPALDKEL<br>KAKGKSALMFNLQEPYFTWPLIAADGGYAFKYENGKYDIKDVGVDNAGAKAGLTFLVDLIKNKHMNAD<br>TDYSIAEAAFNKGETAMTINGPWAWSNIDTSKVNYGVTVLPTFKGQPSKPFVGVLSAGINAASPNKELA<br>KEFLENYLLTDEGLEAVNKDKPLGAVALKSYEEELAKDPRIAATMENAQKGEIMPNIPQMSAFWYAVRT<br>AVINAASGRQTVDEALKDAQTNSSSNNNNNNNNNNLGIEGRISEFYIDTDSVVGDTIIDVSGKKMTIAEF<br>YDSTPDGSHHHHHH
**2P**	SBP-[E34]Int^{C}-Trx-H$_6$	MDEKTTGWRGGHVVEGLAGELEQLRARLEHHPQGQREPGASGGGGSSSEARDWVKRVGGKTSLSV<br>NTYSGEVERKNINYIMKHTVKKRMFKIKAGGKEVIVTADHSVMVKRDGKIIDVKPTEMKQTDRVVKWML<br>TGSHMIEFIEFEIEDLGVMEIDVYDIEVDGNHNFFGNDILVHNSVYLNGTGSDKIIHLTDDSFDTDVLKAD<br>GAILVDFWAHWCGPCKMIAPILDEIADEYQGKLTVAKLNIDHNPGTAPKYGIRGIPTLLLFKNGEVAATK<br>VGALSKGQLKEFLDANLAGSEFRSHHHHHH
**3P**	MBP-Int^{N}[T24]-H$_6$	MKTEEGKLVIWINGDKGYNGLAEVGKKFEKDTGIKVTVEHPDKLEEKFPQVAATGDGPDIIFWAHDRFG<br>GYAQSGLLAEITPDKAFQDKLYPFTWDAVRYNGKLIAYPIAVEALSLIYNKDLLPNPPKTWEEIPALDKEL<br>KAKGKSALMFNLQEPYFTWPLIAADGGYAFKYENGKYDIKDVGVDNAGAKAGLTFLVDLIKNKHMNAD<br>TDYSIAEAAFNKGETAMTINGPWAWSNIDTSKVNYGVTVLPTFKGQPSKPFVGVLSAGINAASPNKELA<br>KEFLENYLLTDEGLEAVNKDKPLGAVALKSYEEELAKDPRIAATMENAQKGEIMPNIPQMSAFWYAVRT<br>AVINAASGRQTVDEALKDAQTNSSSNNNNNNNNNNLGIEGRISEFYIDTDSVVGDTIIDVSGKKMTIAEF<br>YDSTGSHHHHHH
**4P**	MBP-Int^{N}[D22]-H$_6$	MKTEEGKLVIWINGDKGYNGLAEVGKKFEKDTGIKVTVEHPDKLEEKFPQVAATGDGPDIIFWAHDRFG<br>GYAQSGLLAEITPDKAFQDKLYPFTWDAVRYNGKLIAYPIAVEALSLIYNKDLLPNPPKTWEEIPALDKEL<br>KAKGKSALMFNLQEPYFTWPLIAADGGYAFKYENGKYDIKDVGVDNAGAKAGLTFLVDLIKNKHMNAD<br>TDYSIAEAAFNKGETAMTINGPWAWSNIDTSKVNYGVTVLPTFKGQPSKPFVGVLSAGINAASPNKELA<br>KEFLENYLLTDEGLEAVNKDKPLGAVALKSYEEELAKDPRIAATMENAQKGEIMPNIPQMSAFWYAVRT<br>AVINAASGRQTVDEALKDAQTNSSSNNNNNNNNNNLGIEGRISEFYIDTDSVVGDTIIDVSGKKMTIAEF<br>YDGSHHHHHH
**5P**	MBP-Int^{N}[F20]-H$_6$	MKTEEGKLVIWINGDKGYNGLAEVGKKFEKDTGIKVTVEHPDKLEEKFPQVAATGDGPDIIFWAHDRFG<br>GYAQSGLLAEITPDKAFQDKLYPFTWDAVRYNGKLIAYPIAVEALSLIYNKDLLPNPPKTWEEIPALDKEL<br>KAKGKSALMFNLQEPYFTWPLIAADGGYAFKYENGKYDIKDVGVDNAGAKAGLTFLVDLIKNKHMNAD<br>TDYSIAEAAFNKGETAMTINGPWAWSNIDTSKVNYGVTVLPTFKGQPSKPFVGVLSAGINAASPNKELA<br>KEFLENYLLTDEGLEAVNKDKPLGAVALKSYEEELAKDPRIAATMENAQKGEIMPNIPQMSAFWYAVRT<br>AVINAASGRQTVDEALKDAQTNSSSNNNNNNNNNNLGIEGRISEFYIDTDSVVGDTIIDVSGKKMTIAEF<br>GSHHHHHH
**6P**	MBP-Int^{N}[S11]-H$_6$	MKTEEGKLVIWINGDKGYNGLAEVGKKFEKDTGIKVTVEHPDKLEEKFPQVAATGDGPDIIFWAHDRFG<br>GYAQSGLLAEITPDKAFQDKLYPFTWDAVRYNGKLIAYPIAVEALSLIYNKDLLPNPPKTWEEIPALDKEL<br>KAKGKSALMFNLQEPYFTWPLIAADGGYAFKYENGKYDIKDVGVDNAGAKAGLTFLVDLIKNKHMNAD<br>TDYSIAEAAFNKGETAMTINGPWAWSNIDTSKVNYGVTVLPTFKGQPSKPFVGVLSAGINAASPNKELA<br>KEFLENYLLTDEGLEAVNKDKPLGAVALKSYEEELAKDPRIAATMENAQKGEIMPNIPQMSAFWYAVRT<br>AVINAASGRQTVDEALKDAQTNSSSNNNNNNNNNNLGIEGRISEFYIDTDSVVGDTIIDVSGSHHHHHH
**7P**	SBP-[V27]Int^{C}-Trx-H$_6$	MDEKTTGWRGGHVVEGLAGELEQLRARLEHHPQGQREPGASGGGGSSSVFMRRNDEARDWVKRV<br>GGKTSLSVNTYSGEVERKNINYIMKHTVKKRMFKIKAGGKEVIVTADHSVMVKRDGKIIDVKPTEMKQT<br>DRVVKWMLTGSHMIEFIEFEIEDLGVMEIDVYDIEVDGNHNFFGNDILVHNSVYLNGTGSDKIIHLTDDSF<br>DTDVLKADGAILVDFWAHWCGPCKMIAPILDEIADEYQGKLTVAKL<br>NIDHNPGTAPKYGIRGIPTLLLFKNGEVAATKVGALSKGQLKEFLDANLAGSEFRSHHHHHH
**8P**	SBP-[W38]Int^{C}-Trx-H$_6$	MDEKTTGWRGGHVVEGLAGELEQLRARLEHHPQGQREPGASGGGGSSSWVKRVGGKTSLSVNTYS<br>GEVERKNINYIMKHTVKKRMFKIKAGGKEVIVTADHSVMVKRDGKIIDVKPTEMKQTDRVVKWMLTGSH<br>MIEFIEFEIEDLGVMEIDVYDIEVDGNHNFFGNDILVHNSVYLNGTGSDKIIHLTDDSFDTDVLKADGAILV<br>DFWAHWCGPCKMIAPILDEIADEYQGKLTVAKLNIDHNPGTAPK<br>YGIRGIPTLLLFKNGEVAATKVGALSKGQLKEFLDANLAGSEFRSHHHHHH
**9P**	SBP-[G43]Int^{C}-Trx-H$_6$	MDEKTTGWRGGHVVEGLAGELEQLRARLEHHPQGQREPGASGGGGSSSGGKTSLSVNTYSGEVER<br>KNINYIMKHTVKKRMFKIKAGGKEVIVTADHSVMVKRDGKIIDVKPTEMKQTDRVVKWMLTGSHMIEFIE<br>FEIEDLGVMEIDVYDIEVDGNHNFFGNDILVHNSVYLNGTGSDKIIHLTDDSFDTDVLKADGAILVDFWAH<br>WCGPCKMIAPILDEIADEYQGKLTVAKLNIDHNPGTAPKYGIRGIPTLLLFKNGEVAATKVGALSKGQLK<br>EFLDANLAGSEFRSHHHHHH
**10P**	SBP-[G12]Int^{C}-Trx-H$_6$	MDEKTTGWRGGHVVEGLAGELEQLRARLEHHPQGQREPGASGGGGSSSGKKMTIAEFYDSTPDVF<br>MRRNDEARDWVKRVGGKTSLSVNTYSGEVERKNINYIMKHTVKKRMFKIKAGGKEVIVTADHSVMVKR<br>DGKIIDVKPTEMKQTDRVVKWMLTGSHMIEFIEFEIEDLGVMEIDVYDIEVDGNHNFFGNDILVHNSVYL<br>NGTGSDKIIHLTDDSFDTDVLKADGAILVDFWAHWCGPCKMIAPILD<br>EIADEYQGKLTVAKLNIDHNPGTAPKYGIRGIPTLLLFKNGEVAATKVGALSKGQLKEFLDANLAGSEFR<br>SHHHHHH
**12P**	MBP-CLm^{N}-H$_6$	MKTEEGKLVIWINGDKGYNGLAEVGKKFEKDTGIKVTVEHPDKLEEKFPQVAATGDGPDIIFWAHDRFG<br>GYAQSGLLAEITPDKAFQDKLYPFTWDAVRYNGKLIAYPIAVEALSLIYNKDLLPNPPKTWEEIPALDKEL<br>KAKGKSALMFNLQEPYFTWPLIAADGGYAFKYENGKYDIKDVGVDNAGAKAGLTFLVDLIKNKHMNAD<br>TDYSIAEAAFNKGETAMTINGPWAWSNIDTSKVNYGVTVLPTFKGQPSKPFVGVLSAGINAASPNKELA<br>KEFLENYLLTDEGLEAVNKDKPLGAVALKSYEEELAKDPRIAATMENAQKGEIMPNIPQMSAFWYAVRT<br>AVINAASGRQTVDEALKDAQTNSSSNNNNNNNNNNLGIEGRISEFYIDTDSVVGDTIIDVSGKKMTIAEF<br>YDSTPDVFMRRNDEARDWVKRVGGKTSLSVNTYSGEVERKNINYIMKHKVKKRMHKIKAGGKEVIVTA<br>DHSVMVKRDGKIIDVKPTEMKQTDRVVKWNLTGSHHHHHH
**13P**	H$_6$-Smt3-Aes^{C}-sfGFP	MGSSHHHHHHSSGLVPRGSHMASMSDSEVNQEAKPEVKPEVKPETHINLKVSDGSSEIFFKIKKTTPL<br>RRLMEAFAKRQGKEMDSLRFLYDGIRIQADQTPEDLDMEDNDIIEAHREQIGGSMIEFIEFEIEDLGVMEI<br>DVYDIEVDGNHNFFGNDILVHNSVYLNGTSKGEELFTGVVPILVELDGDVNGHKFSVRGEGEGDATNG<br>KLTLKFICTTGKLPVPWPTLVTTLTYGVQCFSRYPDHMKRHDFFKSAMPEGYVQERTISFKDDGTYKTR<br>AEVKFEGDTLVNRIELKGIDFKEDGNILGHKLEYNFNSHNVYITADKQKNGIKANFKIRHNVEDGSVQLA<br>DHYQQNTPIGDGPVLLPDNHYLSTQSVLSKDPNEKRDHMVLLEFVTAAGITHG
**14P**	SBP-Aes^{C}-SBP	MDEKTTGWRGGHVVEGLAGELEQLRARLEHHPQGQREPGASGGGGSSSMIEFIEFEIEDLGVMEIDV<br>YDIEVDGNHNFFGNDILVHNSVYLNGTMDEKTTGWRGGHVVEGLAGELEQLRARLEHHPQGQREP

*Intein fragments are marked in blue (Int^{N}: dark blue, Int^{C}: light blue). The introduction of native extein residues is marked in red. MBP is marked in orange, superfolder GFP in green, Smt3 in violet, SBP in gray and thioredoxin in yellow. Additional residues (e.g., linker, His-tag) are shown in black.

Supplementary Figures

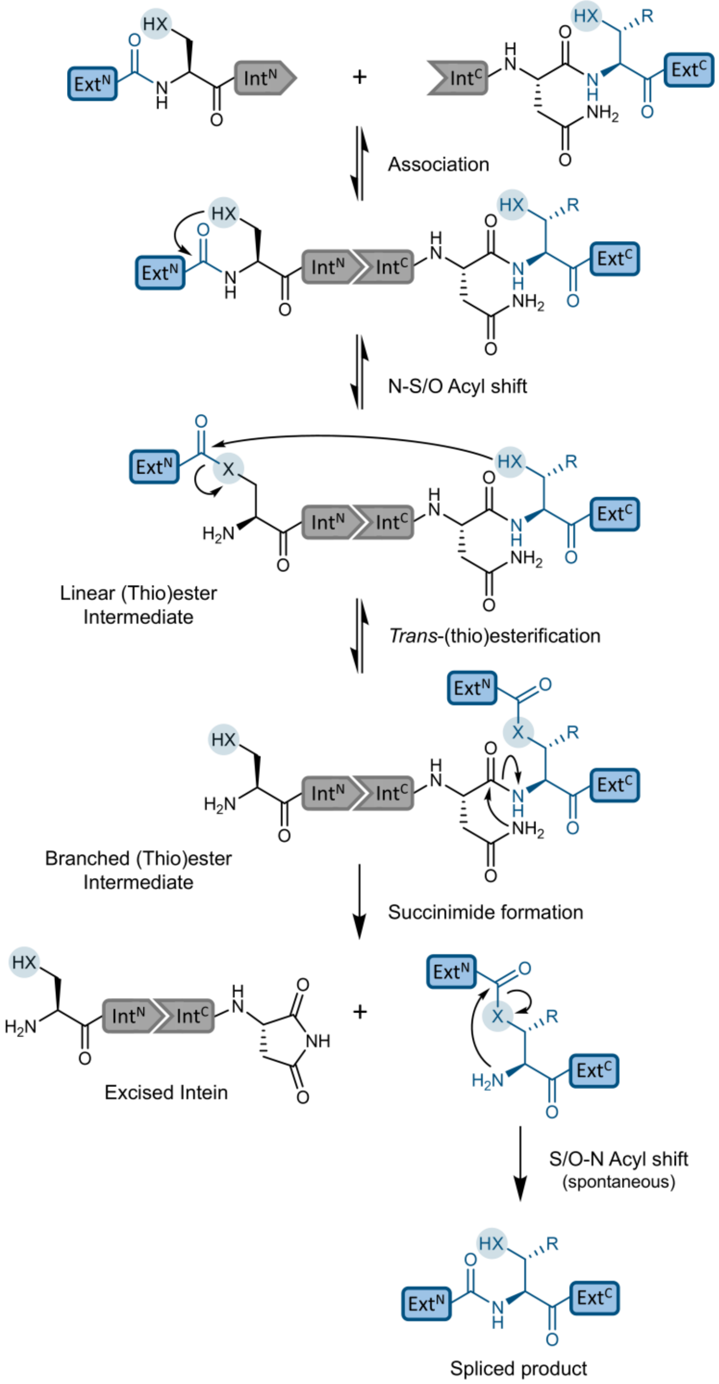

Figure S1. Intein protein splicing mechanism. General protein splicing mechanism of split inteins (X = S, O; R = H, CH_3). Note that cysteine-less inteins operate with Ser1 and Ser+1 (Thr+1) residues at the two splice junctions to form the linear and branched ester intermediates. Initially, an N−S/O acyl shift transfers the N-extein to a Cys or Ser residue at the intein's first position, forming a linear (thio)ester intermediate. In the subsequent trans(thio)esterification step, the Cys, Ser, or Thr side chain from the C-extein's first position (position +1 with regarding to intein numbering) attacks the linear intermediate to form a branched (thio)ester intermediate. The branched intermediate is then resolved by cyclization of a conserved C-terminal asparagine, the last residue of the intein, cleaving the bond between the intein and C-extein, and releasing the intein as a C-terminal succinimide. Finally, the liberated α-amino group of the +1 position triggers a spontaneous S/O−N acyl shift, yielding a native peptide bond between the exteins.

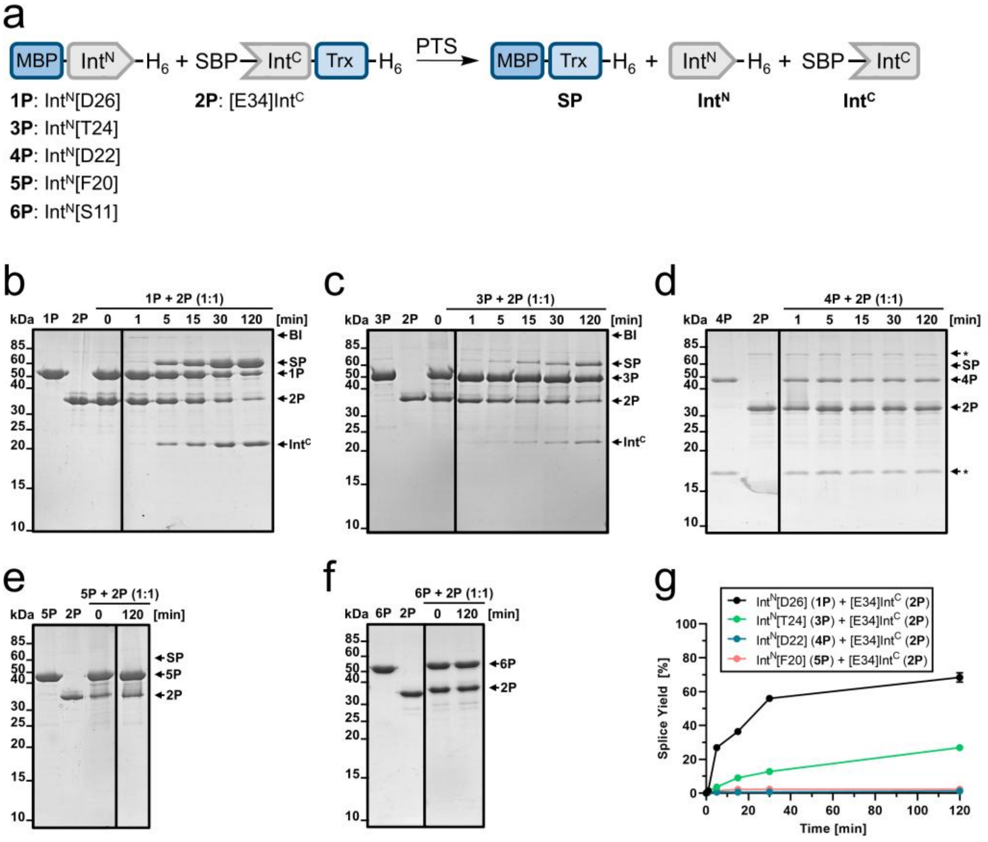

Figure S2. Effects of Int^N shortening on splicing efficiency of the artificially split CL intein. a) Scheme of the PTS reactions. b-f) SDS-PAGE analysis of the PTS reactions shown in (a) using the N-terminal precursors (b) MBP-Int^N[D26]-H_6 (**1P**), (c) MBP-Int^N[T24]-H_6 (**3P**), (d) MBP-Int^N[D22]-H_6 (**4P**), (e) MBP-Int^N[F20]-H_6 (**5P**), and (f) MBP-Int^N[S11]-H_6 (**6P**) together with the C-terminal precursor SBP-[E34]Int^C-Trx-H_6 (**2P**) at equimolar concentrations, at 37 °C. Shown are Coomassie-stained gels. These experiments were performed in duplicate. g) Time-course of the PTS reactions based on densitometric analysis. BI = branched intermediate; MBP = maltose binding protein; SBP = streptavidin binding peptide; SP = splice product; Trx = thioredoxin. For (g), n = 2−3 technical replicates. Data are presented as mean ± SD normalized to the molecular weight of the respective protein species.

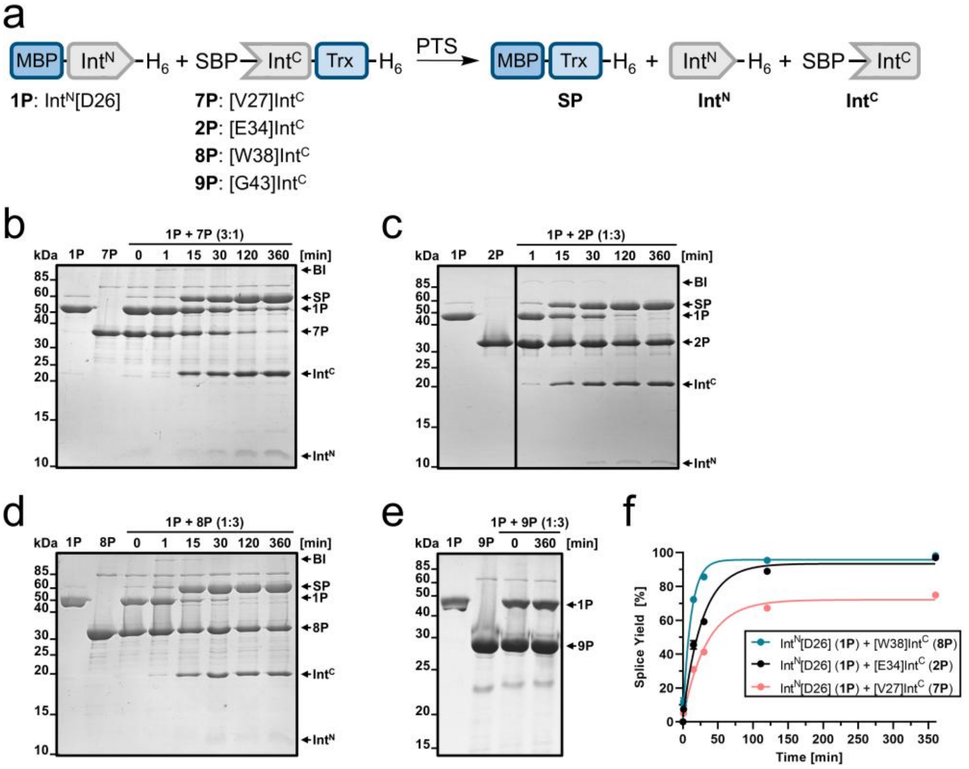

Figure S3. Effects of new split site deletions on the Int^{C} fragment on splicing efficiency of the artificially split CL intein. a) Scheme of the PTS reactions. b-e) SDS-PAGE analysis of the PTS reactions shown in (a) using the N-terminal precursors MBP-Int^{N}[D26]-H_6 (**1P**) together with the C-terminal precursors (b) SBP-[V27]Int^{C}-Trx-H_6 (**7P**), (c) SBP-[E34]Int^{C}-Trx-H_6 (**2P**), (d) SBP-[W38]Int^{C}-Trx-H_6 (**8P**), and (e) SBP-[G43]Int^{C}-Trx-H_6 (**9P**) with one of the precursors given in threefold molar excess as indicated, at 37 °C. Shown are Coomassie-stained gels. These experiments were performed in duplicate or triplicate. g) Time-resolved quantification of splice product formation by densitometric analysis, with data fitted to a one-phase exponential equation. BI = branched intermediate; MBP = maltose binding protein; SBP = streptavidin binding peptide; SP = splice product; Trx = thioredoxin. For (f), n = 2−3 technical replicates. Data are presented as mean ± SD normalized to the molecular weight of the respective protein species.

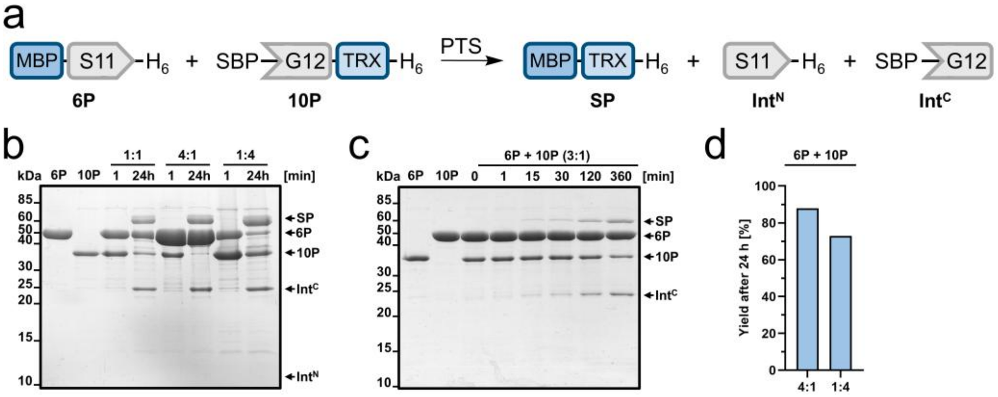

Figure S4. Efficiency of the S11/G12 split site. a) Scheme of the PTS reaction. b-c) SDS-PAGE analysis of the PTS reaction shown in (a) using the N-terminal precursors MBP-Int^{N}[S11]-H_6 (**6P**) together with the C-terminal precursors SBP-[G12]Int^{C}-Trx-H_6 (**10P**) with one of the precursors given in three- or fourfold molar excess as indicated, at 37 °C. Shown are Coomassie-stained gels. The experiment in (c) was performed in triplicate. d) Quantification of splice product formation after 24 h by densitometric analysis of the data shown in (b). MBP = maltose binding protein; SBP = streptavidin binding peptide; SP = splice product; Trx = thioredoxin.

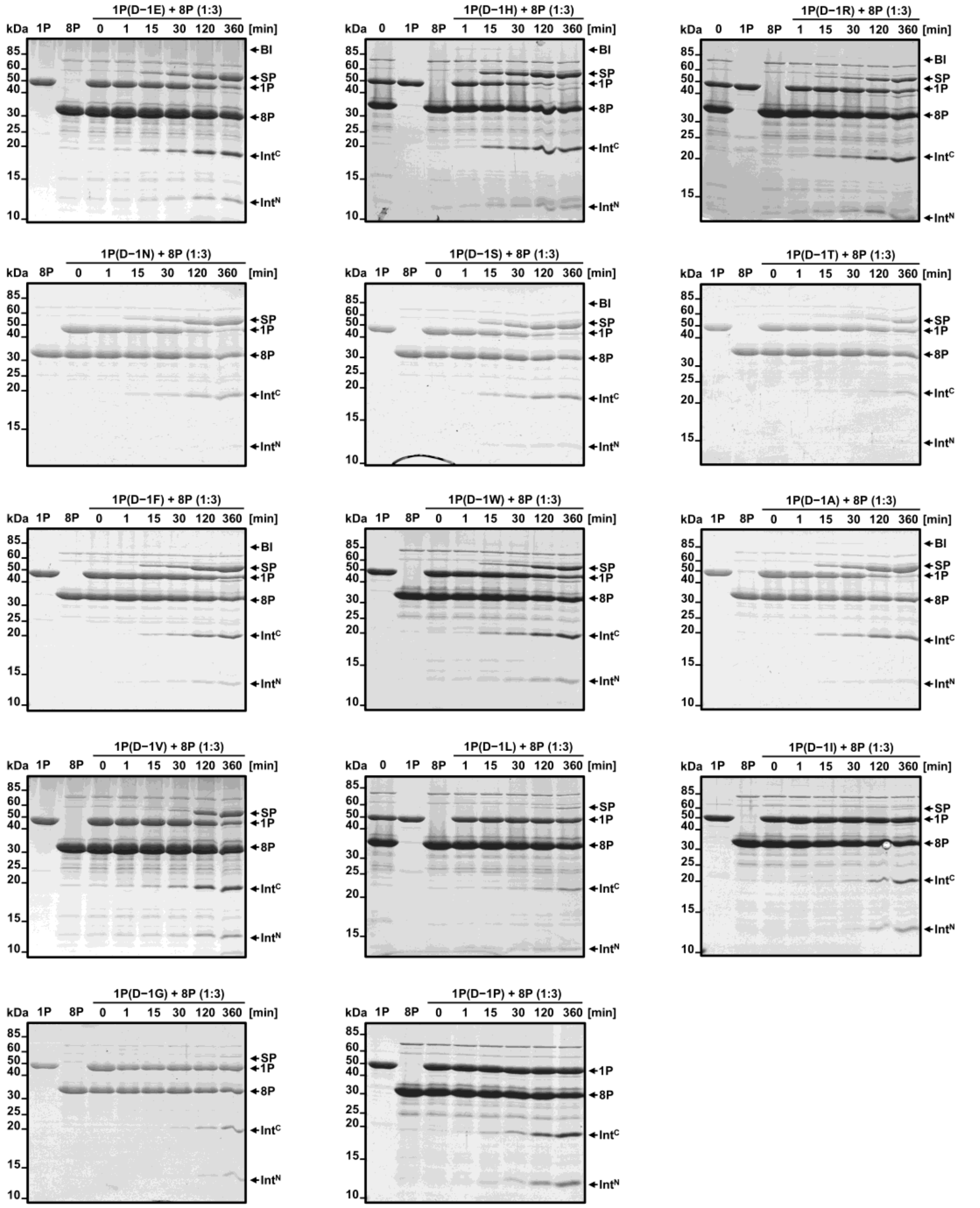

Figure S5. Analysis of the D−1X substitutions introduced into precursor MBP-Int^{N}[D26]-H$_6$ (**1P**) (additional data to Figure 2). Coomassie-stained SDS-PAGE analysis of the PTS reactions using the D−1X substitutions introduced into the N-terminal precursor **1P** with the C-terminal precursor SBP-[W38]Int^{C}-Trx-H$_6$ (**8P**) used in threefold molar excess at 37 °C. These experiments were performed in duplicate. BI = branched intermediate; SP = splice product.

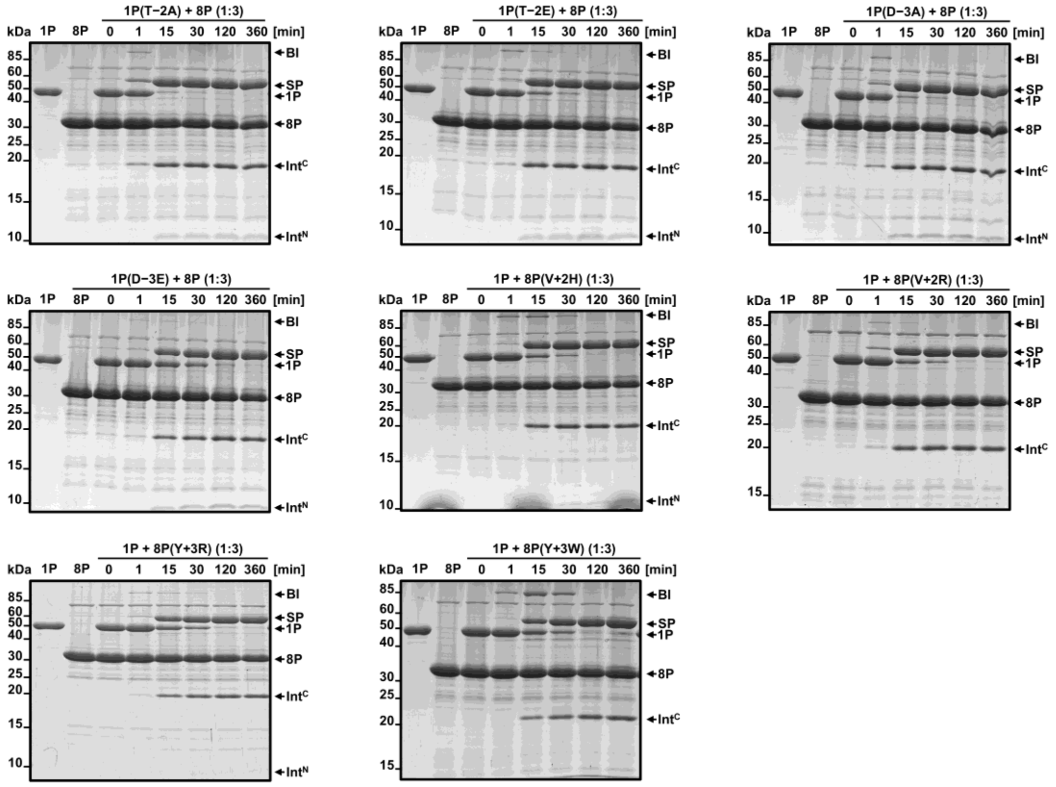

Figure S6. Analysis of the side chain substitutions at the −2, −3, +2, and +3 positions (additional data to Figure 4). Coomassie-stained SDS-PAGE analysis of the PTS reactions using the substitutions introduced into the N-terminal precursor MBP-Int^{N}[D26]-H$_6$ (**1P**) or the C-terminal precursor SBP-[W38]Int^{C}-Trx-H$_6$ (**8P**), respectively. The C-terminal precursor is used in threefold molar excess at 37 °C. These experiments were performed in duplicate. BI = branched intermediate; SP = splice product.

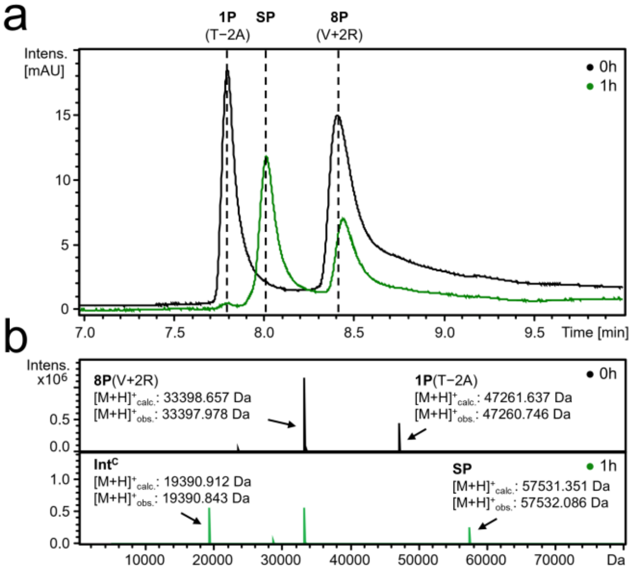

Figure S7. LC-MS analysis of the PTS reaction using **1P**(T−2A) and **8P**(V+2R). Additional data to the PTS reaction analyzed in Figure 5d, e. a) RP-HPLC analysis on a C3 column (ZORBAX StableBond 300 C3, 4.6 x 12.5 mm, 5 µm, Agilent) of the total PTS reaction using **1P**(T−2A) (5 µM) and **8P**(V+2R) (15 µM) at 37 °C. Shown are the UV chromatograms at 280 nm before PTS (black) and 1 h after PTS induction (green). (b) Deconvoluted masses of the ESI-MS analysis after HPLC separation (7.0 – 10.0 min) as shown in (a). SP = splice product.

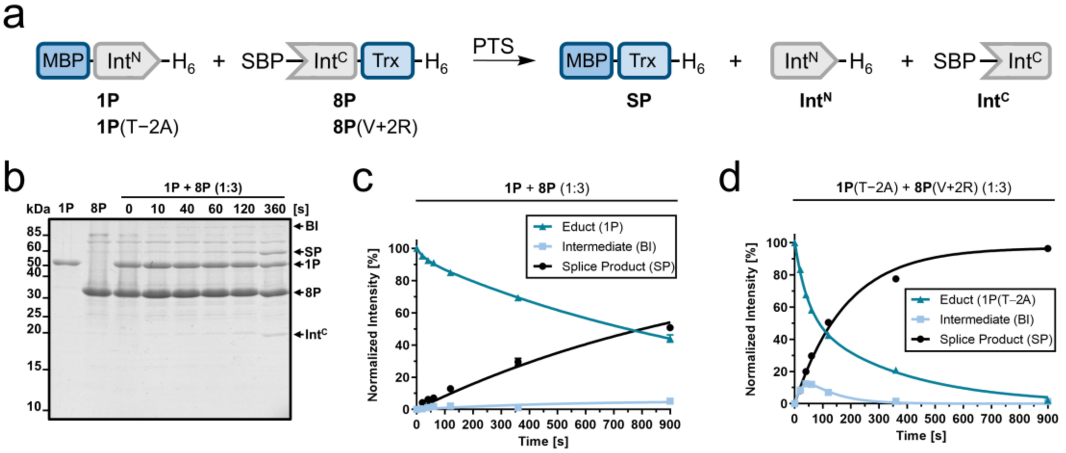

Figure S8. Analysis of the T−2A/V+2R double extein mutation in the CL intein. a) Scheme of the PTS reaction. b) SDS-PAGE analysis of the PTS reaction using the N-terminal precursors **1P** with the C-terminal precursors **8P** used in threefold molar excess at 37 °C. This experiment was repeated two times. Shown is an exemplary Coomassie-stained gel. c) Time-resolved quantification of splice product (**SP**) and branched intermediate (**BI**) formation relative to the precursor **1P** by densitometric analysis, with data fitted to a simplified three-state kinetic model. d) Time-resolved quantification of **SP** and **BI** formation relative to the precursor **1P**(T−2A) by densitometric analysis, with data fitted to a simplified three-state kinetic model (see Method section). BI = branched intermediate; MBP = maltose binding protein; SBP = streptavidin-binding-peptide; SP = splice product; Trx = thioredoxin. For (c-d), n = 2 technical replicates. Data are presented as mean ± SD normalized to the molecular weight of the respective protein species.

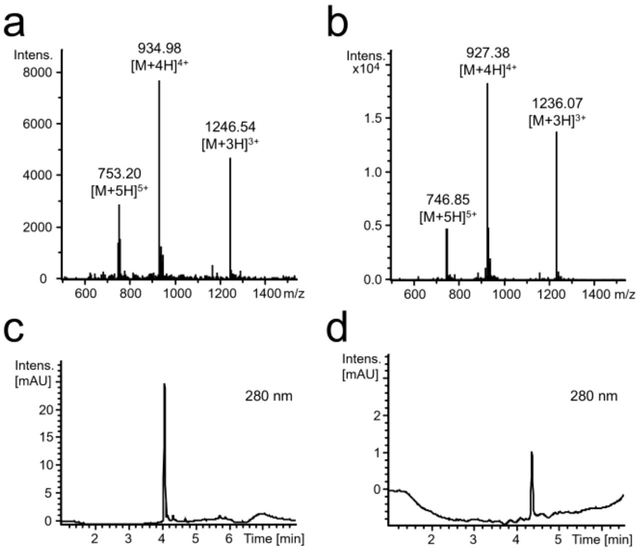

Figure S9. LC-MS analysis of the synthetic peptides **11P** and **11P**(T−2A). a) ESI-MS analysis of the purified synthetic peptide **11P** with M(obs.) 3735.25 Da and M(calc.) 3736.69 Da. b) ESI-MS analysis of the purified synthetic peptide **11P**(T−2A) with M(obs.) 3706.18 Da and M(calc.) 3706.67 Da. c/d) RP-HPLC analysis of the purified synthetic peptides **11P** (c) and **11P**(T−2A) (d) monitored at 280 nm.

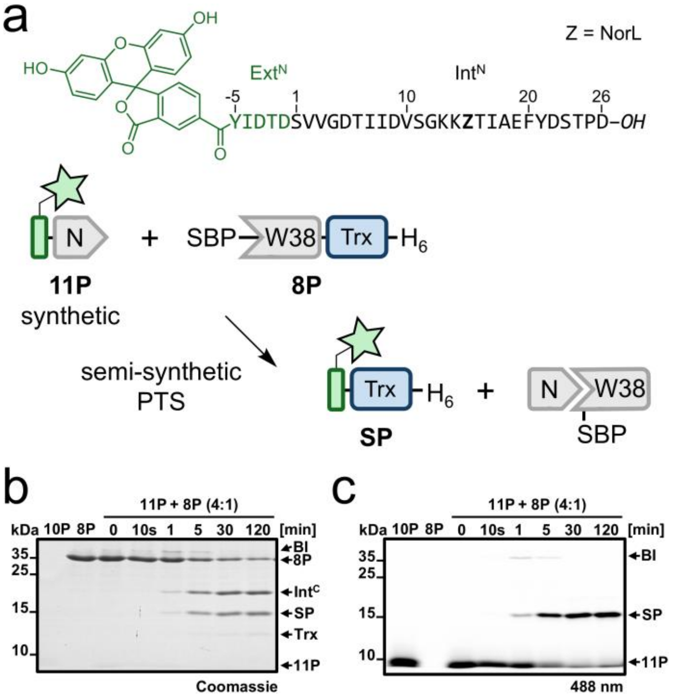

Figure S10. Chemical modification of proteins by semi-synthetic PTS using the optimized CL intein (additional data to Figure 6). a) Sequence of the fluorescein-labeled synthetic peptide Fl-Int^{N}[D26] (**11P**) and scheme of the semi-synthetic PTS reaction with the C-terminal precursor **8P**. Note that M15 was replaced with nor-leucine (Z = NorL) in the synthetic sequence. b/c) SDS-PAGE analysis of the PTS reaction followed by and Coomassie staining (b) and fluorescence imaging (c) using 40 µM **11P** and 10 µM **8P** at 37 °C. This experiment was performed in duplicate. BI = branched intermediate; SP = splice product; Trx = thioredoxin; Ext = extein.

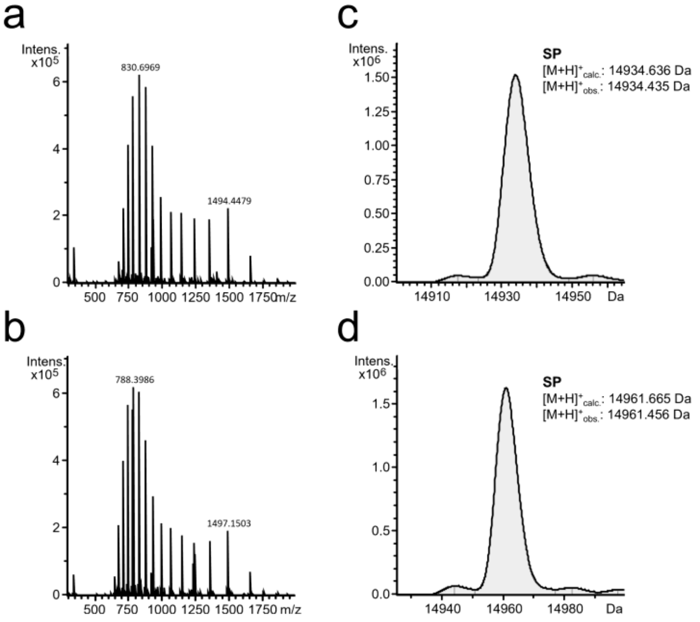

Figure S11. LC-MS analysis of the splice products of the PTS reactions using the synthetic peptides **11P** and **11P**(T−2A). Additional data to the PTS reactions analyzed in Figure 6 and Figure S8. a) MS spectrum of the splice product (**SP**) generated with between **11P** (40 µM) and **8P** (10 µM) at 37 °C. b) MS spectrum of the splice product (**SP**) generated with between **11P**(T−2A) (40 µM) and **8P**(R+2V) (10 µM) at 37 °C. c-d) Deconvoluted masses of the ESI-MS analysis shown in (a) and (b), respectively. SP = splice product.

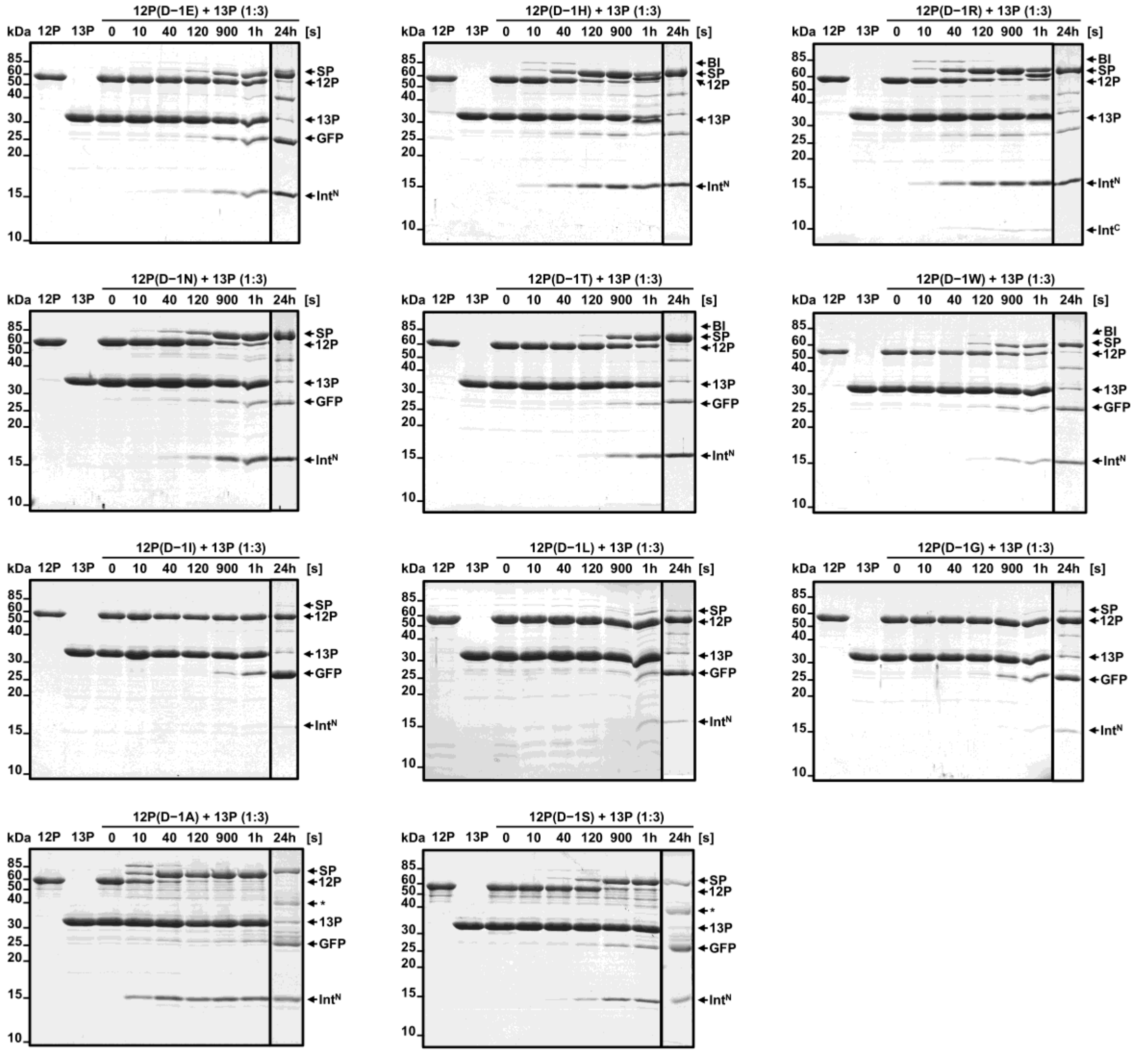

Figure S12. Analysis of the D−1X substitutions introduced into precursor MBP-CLm^N-H$_6$ (**12P**) (additional data to Figure 6). Shown are exemplary Coomassie-stained SDS-PAGE gels to analyze the PTS reactions of the N-terminal precursor **12P** with the indicated D−1X substitutions together with the C-terminal precursor Aes^C-sfGFP (**13P**) used in threefold molar excess at 37 °C. This experiment was performed in duplicate.

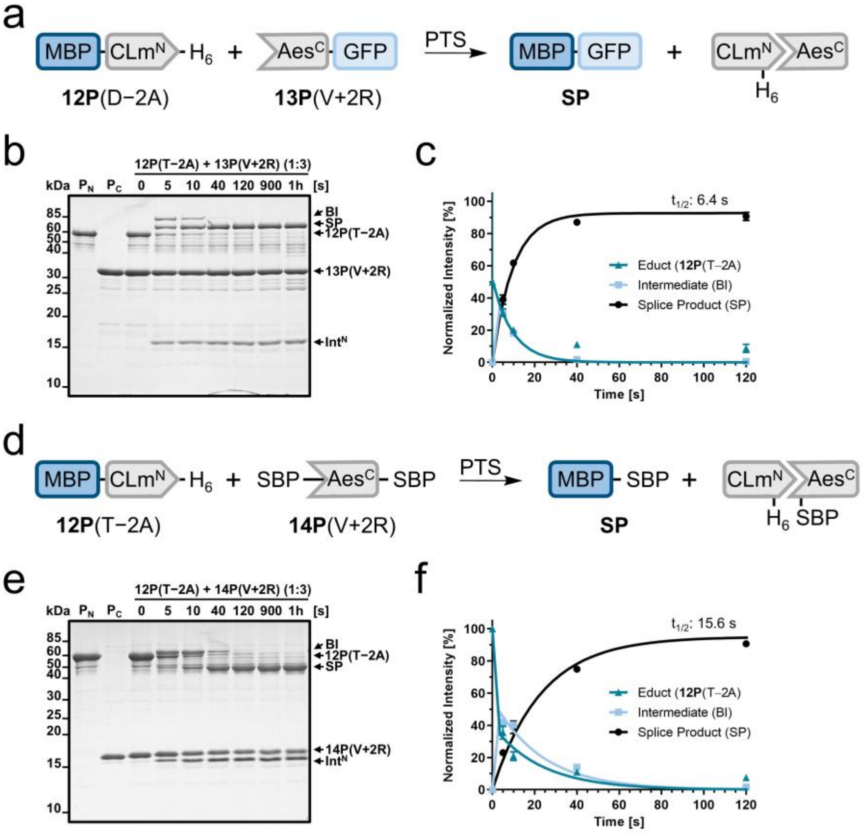

Figure S13. Analysis of the T−2A/V+2R double extein mutation in the CLm intein. a) Scheme of the PTS reaction. b) SDS-PAGE analysis of the PTS reaction shown in (a) using the N-terminal precursors **12P**(T−2A) with the C-terminal precursors **13P**(V+2R) used in threefold molar excess at 37 °C. Shown is an exemplary Coomassie-stained gel. c) Time-resolved quantification of splice product (**SP**) and branched intermediate (**BI**) formation relative to the precursor **12P**(T−2A) by densitometric analysis, with data fitted to a simplified three-state kinetic model (see Method section). d) Scheme of the PTS reaction. e) SDS-PAGE analysis of the PTS reaction shown in (d) using **12P**(T−2A) with the C-terminal precursors **14P**(V+2R) used in threefold molar excess at 37 °C. Shown is an exemplary Coomassie-stained gel. f) Time-resolved quantification of splice product (**SP**) and branched intermediate (**BI**) formation relative to the precursor **12P**(T−2A) by densitometric analysis, with data fitted to a simplified three-state kinetic model. MBP = maltose binding protein. SBP = streptavidin-binding-peptide. For (c, f), n = 2 technical replicates. Data are presented as mean ± SD normalized to the molecular weight of the respective protein species.

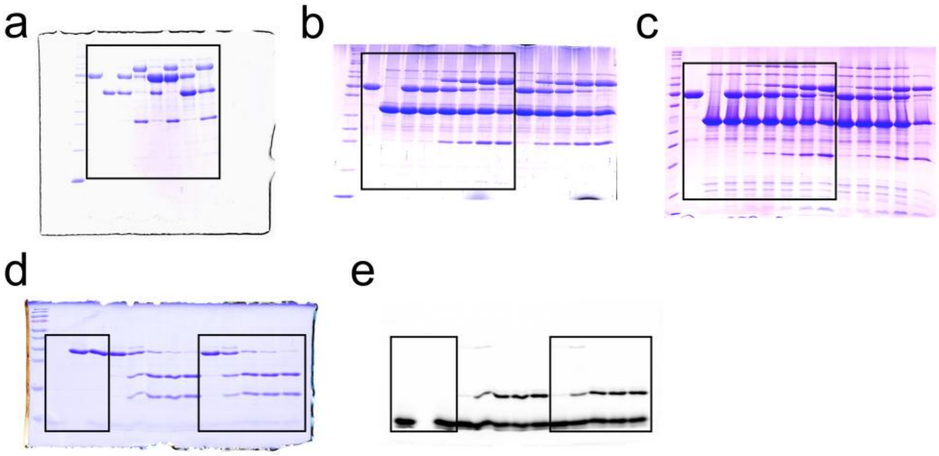

Figure S14. Unprocessed SDS-PAGE images of the figures shown in the main text. The black frame indicates the section used for the figures (a) Figure 2e, (b) Figure 4b, (c) Figure 5d, (d) Figure 6b (upper panel), (e) Figure 6b (lower panel). PageRuler™ unstained protein ladder (Thermo Scientific #26614) or PageRuler™ prestained protein ladder (Thermo Scientific #26616) were used as marker.

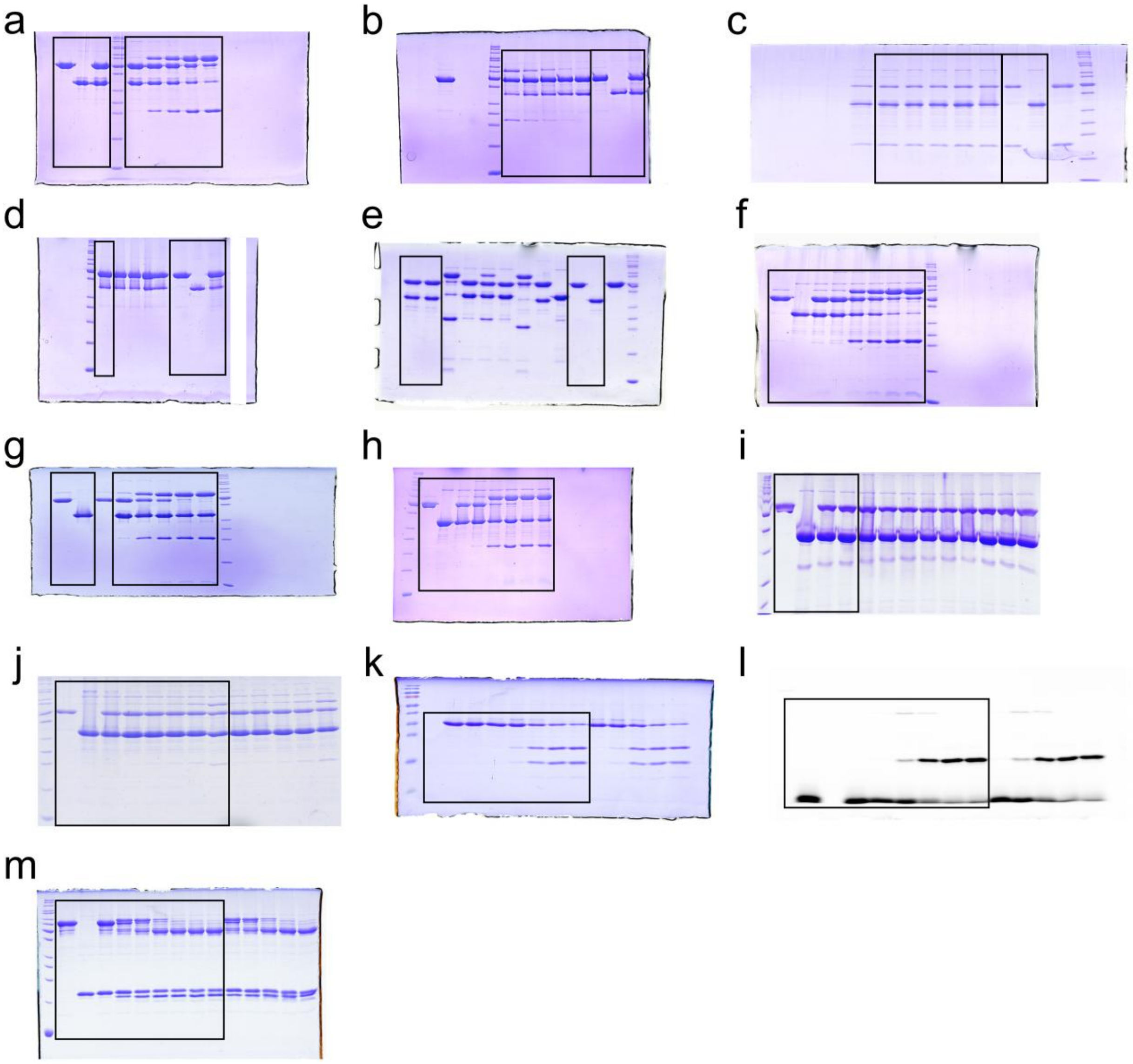

Figure S15. Unprocessed SDS-PAGE images of the figures shown in the supplementary information. The black frame indicates the section used for the figures (a) Figure S2b, (b) Figure S2c, (c) Figure S2d, (d) Figure S2e, (e) Figure S2f, (f) Figure S3b, (g) Figure S3c, (h) Figure S3d, (i) Figure S3e, (j) Figure S7b, (k) Figure S9b, (l) Figure S9c, (m) Figure S12b. PageRuler™ unstained protein ladder (Thermo Scientific #26614) or PageRuler™ prestained protein ladder (Thermo Scientific #26616) were used as marker.

Supplementary References

[1] N. H. Shah, E. Eryilmaz, D. Cowburn, T. W. Muir, *J Am Chem Soc*, **2013**, *135*, 5839–5847.

[2] T. Pasch, A. Schröder, S. Kattelmann, M. Eisenstein, S. Pietrokovski, D. Kümmel, H. D. Mootz, *Chem Sci*, **2023**, *14*, 5204-5213.

[3] M. Bhagawati, T. M. E. Terhorst, F. Füsser, S. Hoffmann, T. Pasch, S. Pietrokovski, H. D. Mootz, *Proc Natl Acad Sci U S A*, **2019**, *116*, 22164–22172.

[4] C. Humberg, Z. Yilmaz, K. Fitzian, W. Dörner, D. Kümmel, H. D. Mootz, *Nat comm*, **2025**, *16*, 2723.
